# Bacterial TANGO2 homologs are heme-trafficking proteins that facilitate biosynthesis of cytochromes *c*

**DOI:** 10.1128/mbio.01320-23

**Published:** 2023-07-18

**Authors:** Sirui Han, Kailun Guo, Wei Wang, Yizhi J. Tao, Haichun Gao

**Affiliations:** 1 Institute of Microbiology, College of Life Sciences, Zhejiang University, Hangzhou, Zhejiang, China; 2 Department of BioSciences, Rice University, Houston, Texas, USA; Michigan State University, Ann Arbor, Michigan, USA; Michigan State University, Ann Arbor, Michigan, USA

**Keywords:** heme trafficking, heme-binding protein, cytochrome *c*, cytochrome *c *maturation, TANGO2

## Abstract

Heme, an essential molecule for virtually all living organisms, acts primarily as a cofactor in a large number of proteins. However, how heme is mobilized from the site of synthesis to the locations where hemoproteins are assembled remains largely unknown in cells, especially bacterial ones. In this study, with *Shewanella oneidensis* as the model, we identified HtpA (SO0126) as a heme-trafficking protein and homolog of TANGO2 proteins found in eukaryotes. We showed that HtpA homologs are widely distributed in all domains of living organisms and have undergone parallel evolution. In its absence, the cytochrome (cyt) *c* content and catalase activity decreased significantly. We further showed that both HtpA and representative TANGO2 proteins bind heme with 1:1 stoichiometry and a relatively low dissociation constant. Protein interaction analyses substantiated that HtpA directly interacts with the cytochrome *c* maturation system. Our findings shed light on cross-membrane transport of heme in bacteria and extend the understanding of TANGO2 proteins.

**IMPORTANCE:**

The intracellular trafficking of heme, an essential cofactor for hemoproteins, remains underexplored even in eukaryotes, let alone bacteria. Here we developed a high-throughput method by which HtpA, a homolog of eukaryotic TANGO2 proteins, was identified to be a heme-binding protein that enhances cytochrome *c* biosynthesis and catalase activity in *Shewanella oneidensis*. HtpA interacts with the cytochrome *c* biosynthesis system directly, supporting that this protein, like TANGO2, functions in intracellular heme trafficking. HtpA homologs are widely distributed, but a large majority of them were found to be non-exchangeable, likely a result of parallel evolution. By substantiating the heme-trafficking nature of HtpA and its eukaryotic homologs, our findings provide general insight into the heme-trafficking process and highlight the functional conservation along evolution in all living organisms.

## INTRODUCTION

Hemoproteins play irreplaceable roles in a myriad of physiological processes, such as oxygen transport, electron transfer, oxidative stress response, respiration, and catalysis (1, 2). Hemoproteins can be divided into two distinct groups depending on the characteristics of heme association: proteins with non-covalently bound heme (Type I) and proteins containing one or more covalently attached heme(s) (Type II) (3). Heme species within Type I hemoproteins include heme *b* (protoheme IX), which is the predominant heme species in the cell and/or the derivatives of heme *b*, such as *a*, *d*, and *o*; on the other hand, heme *b* is attached via two thioether bonds to a heme-binding motif (HBM, typically CXXCH) in Type II hemoproteins, which are collectively called cytochrome (cyt) *c* (4). In addition to its physiological roles, heme also has the potential to cause cytotoxicity, especially when not bound to a protein (5). Therefore, it is crucial to have a tight control of the intracellular pool of total heme, including sequestered heme within hemoproteins and exchangeable heme (often called labile heme) that provides an accessible supply of heme to target proteins (6, 7).

Heme homeostasis within cells is maintained primarily by balancing the rates of heme biosynthesis and catabolism (7, 8). Both processes have been studied intensively for decades, and their metabolic pathways and enzymatic machineries, which are largely conserved in prokaryotes and eukaryotes, have been well established (9 - 11). In contrast, how cells mobilize heme from the site of synthesis to heme-dependent proteins and pathways remains largely unknown. In eukaryotic cells, although it has been proposed that heme can be trafficked through hydrophobic channels without specific carriers, multiple lines of evidence support that heme chaperons are involved in allocating exchangeable heme (8, 12 - 15). Most of these chaperone proteins traffick heme from the mitochondria where the synthesis occurs, to the cytosol and the nucleus, enabling Type I hemoproteins to mature and function.

Bacteria also host a large variety of Type I hemoproteins such as catalases, oxygen reductases (cyt *bd*, *bo*, *aa*_3_, and *ba*_3_), transient heme-binding proteins for heme utilization, oxygenases, cyt P450 enzymes, and heme-dependent regulators (1, 10). Given that bacteria generally lack intracellular organelles and heme is synthesized in the same compartment as Type I hemoproteins, one may imagine that heme trafficking may not be necessary. To date, only one bacterial protein, HemW of *Escherichia coli*, has been suggested to be a heme-trafficking protein based on its interaction with bacterioferritin and nitrate reductase component NarI (16).

Many bacteria are renowned for respiratory versatility, a feature largely attributable to cyts *c*, which are located exclusively outside the cytoplasm and commonly function as electron transfer proteins and/or oxidoreductive enzymes (4, 17). Conceivably, these bacteria generally encode a large number of distinctive cyts *c*, for example, 111 and 42 for *Geobactor sulfurreducens* and *Shewanella oneidensis,* respectively (18, 19). The heme attachment in cyts *c* is through posttranslational modification, called cyt *c* maturation (CCM) or biosynthesis, by one of three dedicated protein systems (4). System I is composed of eight or nine proteins (CcmA-H or CcmA-I), widely found in diverse Gram-negative bacteria and archaea, as well as in plant and protozoan mitochondria (4). In Gram-negative bacteria, heme attachment occurs in the periplasm, requiring transmembrane transport of both apocyt *c* polypeptide and heme (4). It is now known that CcmABC of System I constitutes an ABC transporter specialized for heme transport across the cytoplasmic membrane (20 - 22).

As newly synthesized heme may not be in close proximity of the CCM apparatus, we propose that cells may need heme chaperons to deliver heme to the heme transport complex in bacteria. In this study, we tested this proposal with *S. oneidensis* as a model because of the abundance of cyts *c*, which confers on cells (colony and cell-pellet) a red-brown color, a phenotype that facilitates the screening for genes associated with changes in the cyt *c* content (23 - 26). By exploiting random mutagenesis, we identified SO0126 as a heme-trafficking protein, which not only promotes the efficiency of CCM by directly interacting with System I but also is implicated in trafficking heme to catalase KatB, a Type I hemoprotein. Additionally, we demonstrated that SO0126 is a homolog of eukaryotic TANGO2 (*t*ransport *an*d *G*olgi *o*rganization) proteins, initially identified in *Drosophila melanogaster* (27), and confirmed that TANGO2 proteins of *D. melanogaster* and *Homo sapiens* are heme-binding proteins. By echoing the recent findings that TANGO2 proteins are involved in heme trafficking (28), the presented study complements and extends understanding of TANGO2 proteins.

## RESULTS

### Overexpression of SO0126 elevates the cyt *c* content in *S. oneidensis*

**Fig 1:**
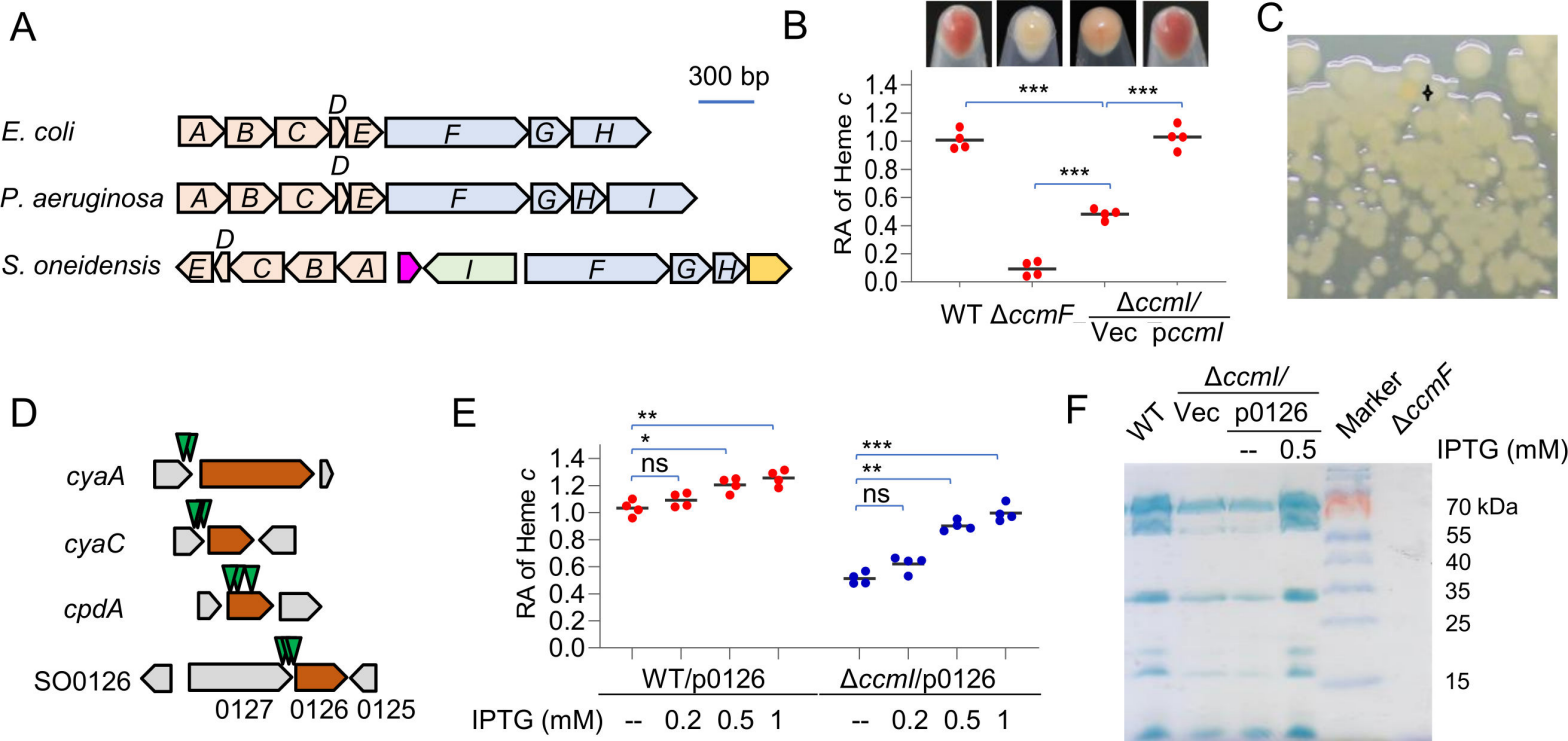
SO0126 in overproduction suppresses the defect caused by the CcmI loss in *S. oneidensis*. (**A**) Organization of the *ccm* genes in representative γ-proteobacteria. The *ccm* genes, regardless of *ccmA-H* as in *E. coli* or *ccmA-I* as in *Pseudomonas aeruginosa*, are commonly organized into a single operon. In *S. oneidensis*, the *ccm* genes are clustered but constitute multiple operons. The genes belonging to the same functional module are shown in the same color. (**B**) The CcmI loss reduces the cyt *c* content and makes cell pellets less red. Shown are pellets of *S. oneidensis* cultures grown on O_2_ as electron acceptor to the early stationary phase, which were subsequently lysed and subjected to heme *c* quantification. Heme *c* content was normalized to the protein concentration. Relative abundance (RA) of heme *c* for each strain was then calculated by normalizing to the averaged heme *c* content in the wild-type (WT). Throughout this study, shown are the data from biological replicates with asterisks indicating statistically significant differences of the values compared (*n* = 4; ns, not significant; *P < 0.05; **P < 0.01; ***P < 0.001) unless otherwise noted. All strains without expressing a plasmid-borne gene carried an empty vector (Vec), and p*ccmI* represents ∆*ccmI* carrying a complementary vector. Cyt *c*-deficient ∆*ccmF* was included as the negative control. (**C**) Screening for suppressors of ∆*ccmI* by transposon mutagenesis based on differences in the colony color. (**D**) Transposon insertion sites identified by genetic mapping. Shown are the sites having transposon insertions indicated by arrows from multiple mutants. The transposable element carries a robust promoter supporting the expression of genes located downstream of the insertion. Genes linked to the cyt *c* abundance confirmed before or in this study are in red. (**E**) The cyt *c* content increases with the increasing expression of SO0126. Expression of SO0126 (p0126) was driven by isopropyl-β-d-thiogalactopyranoside (IPTG)-inducible promoter P*tac*. (**F**) Heme staining. Samples prepared the same as for quantification of heme *c* were processed, and equal amounts of proteins were separated in SDS-PAGE and then were subjected to heme staining.

*S. oneidensis* possesses a highly conserved CCM system (System I) that is composed of CcmABCDEFGHI (23, 29). The CCM components of *S. oneidensis* are encoded by three operons, *ccmABCDE* (heme transport and delivery), *ccmI*, and *ccmFGH* (heme-apocyt *c* ligation) in the *ccm* locus, different from the organization in most other γ-proteobacteria where the *ccm* genes constitute a single operon (20 - 23) (Fig. 1A). CcmI in *S. oneidensis* is not absolutely required for the maturation of cyts *c* with the canonical HBM (CXXCH). Compared to the wild type (WT), the absence of CcmI (∆*ccmI*) resulted in approximately 50% reduction in the overall cyt *c* abundance, deduced from the cellular level of heme *c* (Fig. 1B). Heme *c* quantification was carried out on the proteome that was extracted from cells grown to the early stationary phase, denatured to remove non-covalently bound heme, and precipitated. The reduction in the overall cyt *c* abundance caused by the *ccmI* deletion could be readily detected by the less red color of the mutant colonies and cell pellets (23) (Fig. 1B). Based on these observations, we reasoned that we could identify critical proteins that, when present at altered levels, affect the efficiency of the CCM system by screening a random mutation library of the ∆*ccmI* strain for colonies visually distinguishable (by color) from the parental ones.

To generate this random mutation library, we employed mariner-based transposon vector pHGT01, which has a robust promoter embedded in the transposable sequence (30). From ~40,000 colonies formed on lysogeny broth (LB) agar plates supplemented with proper antibiotics under aerobic conditions, 32 were found to differ in color (Fig. 1C). Among them, eight isolates had the transposon inserted multiple times in the regions upstream of adenylate cyclase genes *cyaA* and *cyaC* and in the coding sequence of the *cpdA* gene, which encodes a cAMP phosphodiesterase (Fig. 1D). As CyaA/CyaC and CpdA catalyze synthesis and degradation of cAMP, respectively, and cAMP at elevated concentrations enhances the cyt *c* abundance in *S. oneidensis* (31), these insertions are fully justified (32, 33).

Three isolates had transposon insertions mapped near the end of the SO0127 coding sequence and in the upstream region of SO0126 (Fig. 1D). SO0127 and SO0126 are predicted to encode a metal transporter and a domain of unknown function 833 (DUF833) family protein, respectively. Although these two genes are only 63 bp apart and such an arrangement is strictly conserved in *Shewanella* (Fig. S1A), they are transcribed independently of each other (Fig. S1B) and, therefore, may not be functionally linked.

Similar to *cya* genes, SO0126 was not found to be interrupted by the transposon insertions, implying that its expression rather than function is likely affected by the insertions. To verify this, SO0126 was expressed at varying levels driven by IPTG-inducible promoter P*tac* (24), and its effects on the cyt *c* content in the *ccmI* mutant and WT were assessed (Fig. 1E). With IPTG at 1 mM, the cyt *c* content in the ∆*ccmI* strain became comparable to that of WT, validating the suppressing role of SO0126 in the cyt *c* defect resulting from the CcmI loss. The cyt *c* abundance became greater with increasing IPTG levels in both WT and ∆*ccmI* strains, suggesting that the SO0126 protein likely functions independent of CcmI. Moreover, we examined whether the observed impact of SO0126 on the overall cyt *c* content was due to substantially altered production of a subset of cyts *c*. By using heme staining, we found that the cyt *c* profile of the ∆*ccmI* strain expressing SO0126 with 0.5 mM IPTG was similar to that of WT (Fig. 1F), indicating that SO0126 likely affects the production of the entire set of cyts *c* rather than some individual ones. Collectively, these data led us to conclude that SO0126, when overproduced, suppresses the defect in cyt *c* biosynthesis resulting from the depletion of CcmI in *S. oneidensis*.

### SO0126 in excess improves cyt *c* biosynthesis not by replacing CcmI

**Fig 2:**
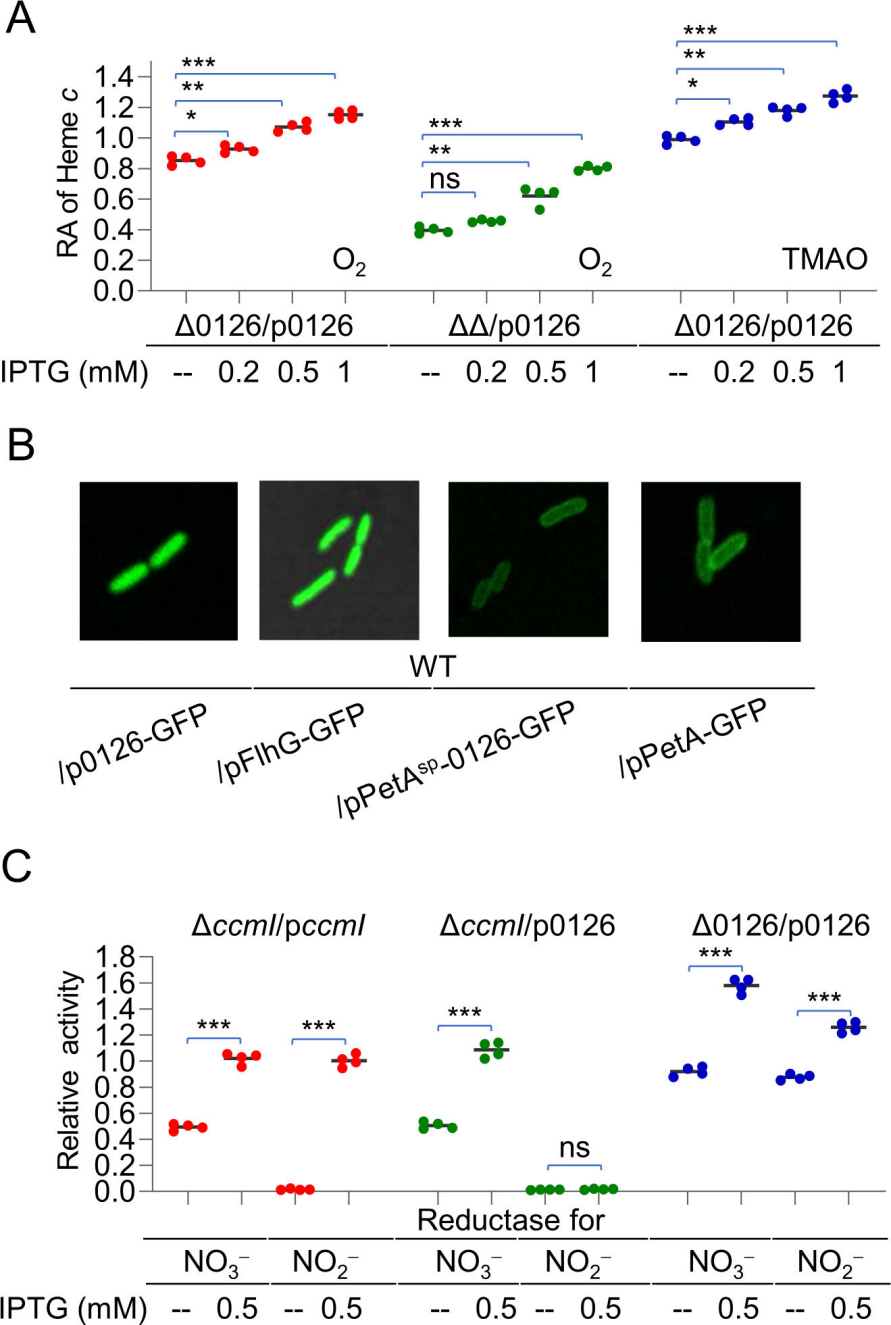
Physiological characterization of SO0126 in *S. oneidensis*. (**A**) The increase in the cyt *c* content with increasing expression of SO0126 is independent of CcmI and electron acceptors. ∆∆ represents ∆*ccmI*∆SO0126. Cultures of the early stationary phase grown on O_2_ or TMAO as the sole electron acceptor were collected, pelleted, and subjected to heme *c* quantification. The data were processed and are presented as in Fig. 1B. (**B**) Cellular localization of SO0126. WT containing the SO0126-GFP and PetA^sp^-SO0126-GFP fusion proteins was grown to the mid-exponential phase and visualized with a confocal microscope. FlhG and PetA, verified cytoplasmic and inner-membrane proteins, respectively, were used as the control. PetA^sp^-SO0126-GFP represents the fusion protein with the PetA signal peptide at the N-terminus and green fluorescent protein (GFP) at the C-terminus. Shown are representative results. (**C**) SO0126 could not function as CcmI. Nitrate and nitrite at 5 mM were added to the cultures prepared anaerobically, and the changes in concentrations at multiple time points were determined. The averaged levels of enzyme activity in the mutants were calculated and normalized to that in WT, giving to relative activity.

In order to uncover the role of SO0126, an in-frame deletion mutant for the gene was constructed and characterized. The loss of SO0126 introduced a modest, but significant, reduction (~15%) in the cyt *c* content compared to WT (Fig. 2A). A similar effect of the SO0126 loss on the cyt *c* content was also observed in the CcmI-deficient background (Fig. 2A). In both cases, the defects resulting from the depletion of SO0126 could be corrected by the expression of the gene *in trans*, verifying that the observed phenomena are, indeed, due to the deletion (Fig. 2A). When overproduced, SO0126 elevated the cyt *c* content significantly in a CcmI-independent manner. Similar results were also obtained from cells grown under anaerobic conditions, with trimethylamine N-oxide (TMAO) as the sole electron acceptor, suggesting that the effect of SO0126 on the cyt *c* content is independent of electron acceptors used to support growth (Fig. 2A).

Unlike CcmI which is a transmembrane protein (23), SO0126 has neither a signal peptide nor a transmembrane segment as predicted by using TPOCONS (34) (Fig. S2A), suggesting that the protein resides in the cytoplasm. This prediction was confirmed by using two SO0126-GFP recombinant proteins, which differ from each other in whether a signal peptide is present or not. With verified cytoplasmic FlhG and inner-membrane protein PetA of *S. oneidensis* as the control (35, 36), we confirmed that the SO0126-GFP fusion proteins were located in the cytoplasm and the addition of the signal peptide of PetA drove the fusion to the membrane (Fig. 2B). Thus, it is unlikely that SO0126 is able to play the same role as CcmI in cyt *c* biosynthesis.

For further confirmation, we compared the activities of nitrate and nitrite reductases in the ∆*ccmI* strain with or without SO0126 overexpression. In *S. oneidensis*, the activity of the nitrate reductase is correlated with the overall cyt *c* content, whereas CcmI is absolutely essential for the maturation of nitrite reductase NrfA because it carries a non-canonical HBM, CXXCK (23). As expected, the ∆*ccmI* strain exhibited lowered nitrate-reducing activity which was approximately 50% relative to WT but was completely unable to reduce nitrite (Fig. 2C). While the genetic complementation fully corrected the defects of the *ccmI* mutant in both reducing activities, expression of the SO0126 gene enabled the mutant to reduce nitrate like WT but did very little to improve its nitrite-reducing capacity. However, when SO0126 was expressed with 0.5 mM IPTG in the SO0126 mutant, abilities of cells to reduce both nitrate and nitrite were enhanced (Fig. 2C). These results conclude that SO0126 improves cyt *c* biosynthesis by increasing cyt *c* abundance as a whole and not by acting as a functional replacement of CcmI.

### SO0126 homologs are cross-domain distributed and are TANGO2 in some eukaryotes

**Fig 3:**
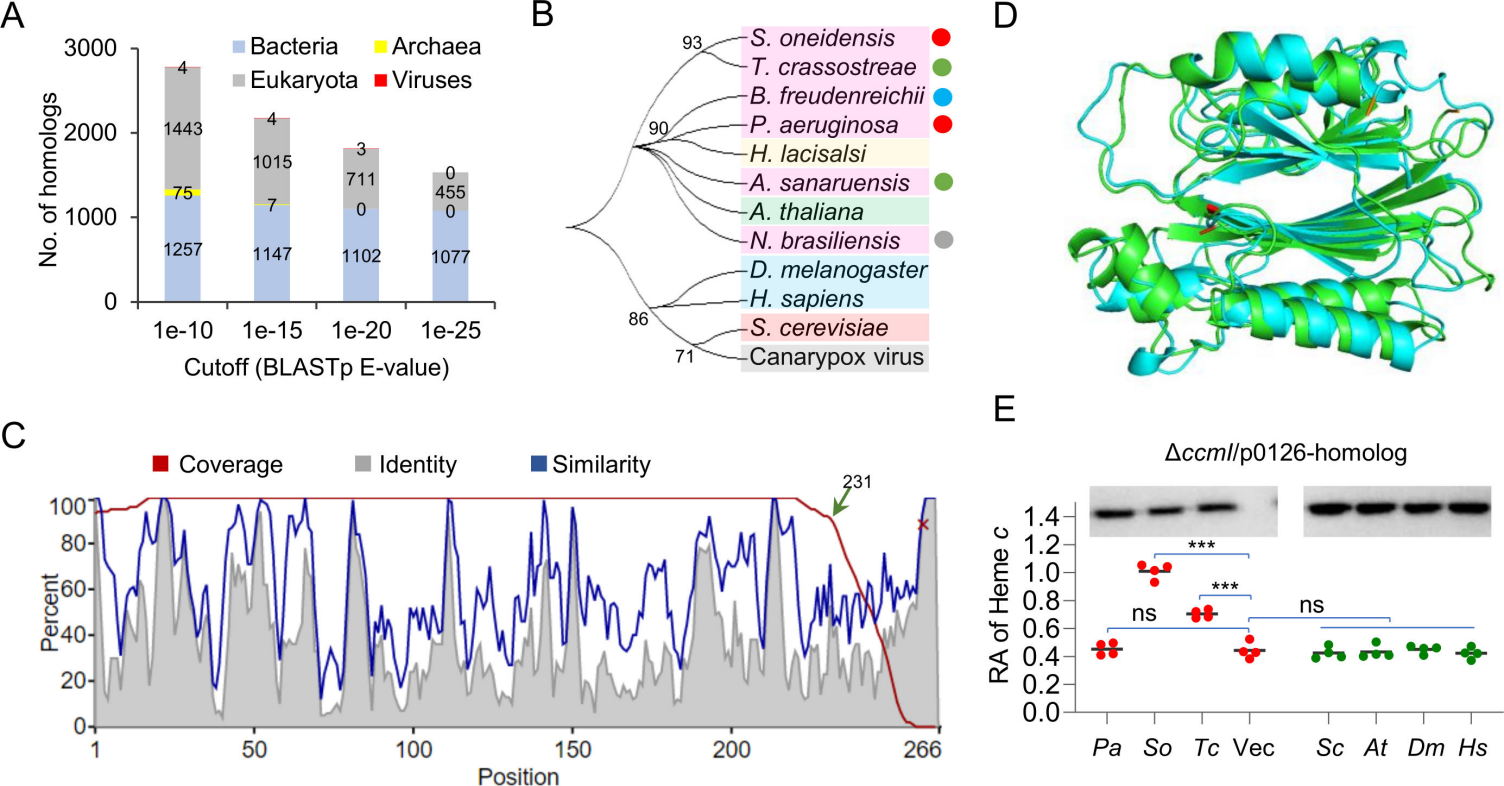
Cross-domain distribution and evolution of SO0126 homologs. (**A**) SO0126 homologs are widely distributed. The numbers of SO0126 homologs in each domain for cellular organisms and viruses with different BLASTp *E*-values as the cutoff. The analysis was carried out with HMMER. (**B**) Phylogenetic analysis of homologs from representative organisms. Bootstrap consensus tree with 50% cutoff was constructed by the neighbor-joining statistical method with Poisson model as the substitution model (Bootstrap replications, 1,000). Bacteria, pink-purple background; Archaea (*Halobiforma lacisalsi*), yellow background; Animalia (*D. melanogaster* and *H. sapiens*), blue background; Plantae (*Arabidopsis thaliana*), green background; Fungi (*Saccharomyces cerevisiae*), red background; Viruses, gray background. Four bacterial phyla: Pseudomonadota (*S. oneidensis* and *P. aeruginosa*, red dots), Bacillota (*Bacillus freudenreichii*, blue dot), Bacteroidota (*Tenacibaculum crassostreae* and *Algoriphagus sanaruensis,* green dots), and Actinomycetota (*Nocardia brasiliensis,* gray dot). (**C**) Hit coverage and similarity graph of *H. sapiens* TANGO2 and SO0126. The analysis was conducted with HMMER. (**D**) Structure comparison of *H. sapiens* TANGO2 (green) and SO0126 (blue). The N-terminus and C-terminus were marked in red and orange, respectively. The structures were predicted with AlphaFold2, and the superimposed comparison was operated with PyMOL. (**E**) Most of SO0126 homologs fail to act as SO0126 in *S. oneidensis*. The ∆*ccmI* strain was complemented with indicated homologs carrying Strep-tag II, whose expression was demonstrated by Western blots (representative results). Cells grown on O_2_ as electron acceptor to the mid-exponential phase, induced with 1 mM IPTG for 10 h, were collected for Western blots. The mouse anti-Strep-tag-II monoclonal antibody (1:5,000 dilution) and the goat anti-mouse IgG-HRP (1:100,000 dilution) were used. The same cultures were subjected to heme *c* quantification. *P. aeruginosa* (*Pa*), *S. oneidensis* (*So*), *T. crassostreae* (*Tc*), *S. cerevisiae* (*Sc*)*, A. thaliana* (*At*)*, D. melanogaster* (*Dm*), and *H. sapiens* (*Hs*).

To get a better understanding of SO0126, we analyzed the protein sequence with multiple bioinformatics programs, including BLASTp, HMMER, and ALPHA-FOLD (37, 38). With the cutoff set to a BLASTp *E*-value of 1e-10, HMMER analysis returned thousands of homologous proteins from organisms in all three domains and even viruses, indicating that SO0126 homologs are extremely widespread (Fig. 3A). While the total number of homologs decreased conversely with *E*-values, bacterial and eukaryotic homologs prevailed in all returned data sets, with homologs from archaea and viruses disappearing when 1e-20 and 1e-25 were used as the cutoff, respectively. A phylogenetic analysis of the homologs from representative organisms revealed an unrooted tree with only four statistically confident linkages, including one that contains *S. oneidensis*/*Tenacibaculum crassostreae*, indicating that these proteins have largely undergone parallel evolution (Fig. 3B). Thus, it is nearly impossible to define the evolutionary relationships as the common ancestors are largely absent and substantial conflict across the tree is widespread. A phylogenetic analysis of bacterial homologs only also supported parallel evolution (Fig. S3), suggesting that the main cause of conflict is due to a biological process hybridization rather than incomplete lineage sorting or a lack of informative characters. Notably, SO0126 homologs are missing in the order of enterobacteriales, which contain some of the best-studied model bacteria such as *Escherichia* and *Salmonella*.

In spite of thousands of SO0126 homologs found in bacteria, there has not been any report of investigations of these proteins. However, eukaryotic homologs of SO0126 from animals, which are called TANGO2, were recently demonstrated to be heme-binding proteins that function in intracellular heme trafficking to proteins (28). To obtain an overview of how the ensemble of *H. sapiens* TANGO2 matches SO0126, a hit coverage and similarity graph were generated with HMMER (37) (Fig. 3C). Although the sequence similarity and identity vary substantially throughout the polypeptide, multiple regions are highly conserved with over 60% identity between the two proteins. In addition, the overall positional match information, called coverage, remains rather high for the entire TANGO2 domain (a.a. 1-231) of the *H. sapiens* protein (276 a.a.), suggesting a strong functional linkage.

The predicted structure of *H. sapiens* TANGO2, available in the AlphaFold Protein Structure Database (38), shows an overall globularly shaped structure made of two central β-sheets with four peripheral α-helices (Fig. S2B). The β-sheets, each of which contains six anti-parallel β-strands, are packed together through a hydrophobic core. Each β-sheet is further surrounded by a set of two extended α-helices from the side facing the exterior. In addition, there are three small α-helices near the C-terminus of the polypeptide. For comparison, the structure of SO0126 was predicted by AlphaFold2 and superimposed onto that of the human TANGO2 (Fig. 3D). Apparently, these two proteins share a high level of structural similarity, especially in the region of the β-sheets and α-helices.

To test whether SO0126 and its homologs are functionally exchangeable, we expressed six representatives in the Δ*ccmI* strain. These selected proteins are from γ-proteobacteria *T. crassostreae* and *P. aeruginosa*, which are in the same phylogenetic clades with *S. oneidensis* or not, respectively, as well as from eukaryotes *Saccharomyces cerevisiae*, *Arabidopsis thaliana*, *D. melanogaster*, and *H. sapiens* (Fig. 3B). Western blots validated the expression of all the proteins in the cells (Fig. 3E). While the SO0126 homolog of *T. crassostreae* partially complemented the defect in the cyt *c* content caused by the CcmI loss, all others failed to elicit any detectable effect (Fig. 3E). These data suggest that only the most closely related homologs can replace SO0126 in the cyt *c* biosynthesis of *S. oneidensis*.

### SO0126 and TANGO2 are heme-binding proteins

In *S. oneidensis*, both apocyt *c* and heme need to cross the cytoplasmic membrane for cyt *c* biosynthesis in the periplasm. It has been established that the general secretion (Sec) pathway is fully and exclusively responsible for the apocyt *c* transport across the cytoplasmic membrane (4, 39). We, therefore, hypothesized that SO0126 promotes cyt *c* biosynthesis by either enhancing heme biosynthesis or delivering newly synthesized heme to the heme transport module of the CCM apparatus, CcmABC. The first possibility appeared unlikely as the expression of rate-limiting enzymes in the heme biosynthetic pathway and intracellular heme levels was not significantly affected by SO0126 overexpression (Fig. S4A and S4B).

**Fig 4:**
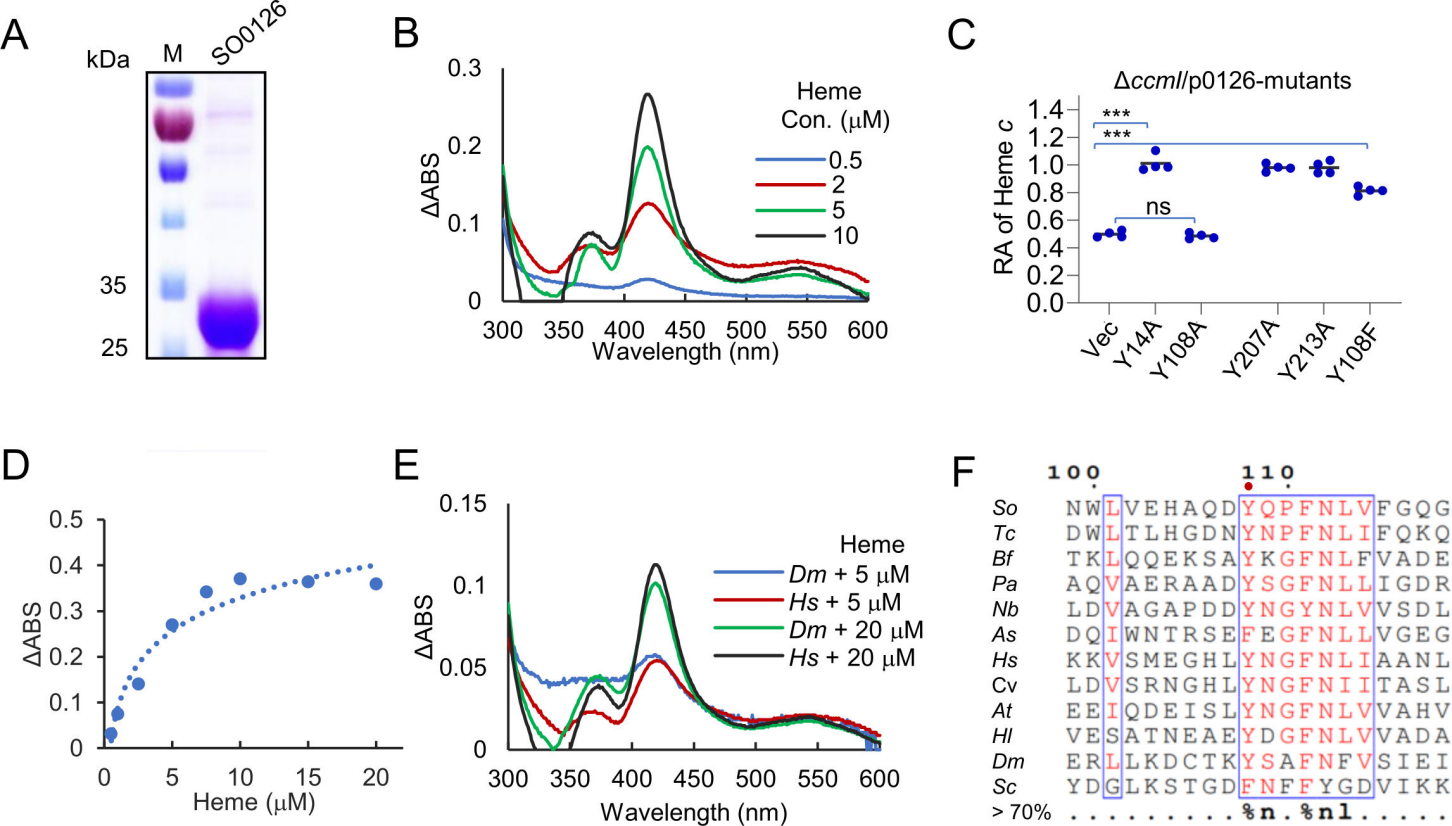
SO0126 and its eukaryotic homologs are heme-binding proteins. (**A**) Expression and purification of recombinant SO0126 with Strep-tag II. Recombinant SO0126 with Strep-tag II was expressed in the SO0126 mutant, purified, and subjected to SDS-PAGE. M, molecular mass standard. SO0126 represents SO0126 protein with Strep-tag II. (**B**) Subtractive UV-visible (UV-vis) spectra of SO0126. In the cuvette, 10 µM purified SO0126, 10 µM hemin, w/o 5 mM DTT were incubated in the dark at 20°C up to 6 h, and the absorption spectra from 300 to 600 nm were recorded. (**C**) The cyt *c* content in Δ*ccmI* overexpressing SO0126 variants. IPTG at 1 mM was used to overexpress SO0126 variants. Please refer to Fig. S5B for the results of more variants. (**D**) Determination of binding stoichiometry of SO0126 and heme. Up to 30 µM hemin with 5 mM DTT was mixed with 10 µM purified SO0126 in binding reaction solutions and scanned at 412 nm. The data were presented as the average of four replicates. (**E**) Subtractive UV-visible spectra of TANGO2 proteins of *D. melanogaster* and *H. sapiens*. The experiment was performed the same as in (**B**). (**F**) Sequence alignments for the Tyr^108^ region of representative SO0126 homologs. Tyr^108^ (marked by a red dot) is a conserved heme-binding residue. *So, S. oneidensis; Tc*, *T. crassostreae; Bf, B. freudenreichii; Pa*, *P. aeruginosa; Nb*, *N. brasiliensis; As*, *A. sanaruensis; Hs*, *H. sapiens*; Cv, Canarypox virus; *At*, *A. thaliana; Hl*, *H. laacisalsi; Dm*, *D. melanogaster*; and *Sc, S. cerevisiae*.

To characterize SO0126 biochemically, we attempted to express and purify recombinant SO0126 from *E. coli*. However, regardless of the tags used, the protein expressed remained insoluble. At last, using the ΔSO0126 strain as the host, we succeeded in purifying a recombinant SO0126 carrying a Strep-tag II, which has a molecular weight of 31.09 kDa (Fig. 4A). The recombinant SO0126 appeared to be free of heme because it was colorless, contrasting heme-bound proteins which are reddish-brown. Upon mixing SO0126 with hemin, a Soret band at ~412 nm in the absorption spectra, the signature of heme-binding proteins, was observed only after prolonged incubation (i.e., >4 h). The absorption spectra also appeared to be noisy (Fig. S5A), suggesting that the recombinant SO0126 has a limited capability of interacting with hemin or the interaction is not stable. When dithiothreitol (DTT) was included in the mixture, the Soret band at ~412 nm appeared more quickly and was able to reach a higher peak intensity (Fig. 4B). These data indicate that the binding of SO0126 to hemin (in the oxidized state) is much slower and less stable than that to heme (in the reduced state).

To provide additional support that SO0126 is a heme-binding protein, we analyzed the protein for amino acid residues that could be critical for binding heme. Common residues involved in heme-binding include cysteine, tyrosine, and histidine (40). Based on the predicted structure of SO0126, a total of 14 cysteine, tyrosine, and histidine residues located on the protein surface were subjected to alanine-scan analysis. Each of these SO0126 variants was expressed in the Δ*ccmI* strain, and the effect on the cyt *c* content was assessed. The results showed that SO0126^Y108A^ failed to elevate the cyt *c* content, whereas all others behaved like the WT protein (Fig. 4C; Fig. S5B), suggesting that Tyr^108^ is essential for the heme-binding activity. Recombinant SO0126^Y108A^ was then expressed, purified, and incubated with heme. Even after extended incubation, the Soret band at ~412 nm did not appear (Fig. S5A), validating that the mutant protein is unable to bind heme.

To determine the binding stoichiometry of SO0126 and heme, increasing amounts of heme were titrated into 10 µM protein solution and the UV-vis spectra were recorded. The results showed that the absorption at ~412 nm reached the maximum with ~10 µM heme, supporting that SO0126 binds to heme with a 1:1 stoichiometry (Fig. 4D). The binding characteristics of SO0126 to heme were further assessed by employing the tryptophan fluorescence quenching method (Fig. S5C). To obtain the equilibrium dissociation constant (*K*_d_) of SO0126 for heme, the data of the quenching assay were fitted to a single binding isotherm with the equation (*F*_0_ − *F*)/(*F − F*_max_) = [heme]/*K*_d_, and a *K*_d_ of 1.2 ± 0.4 µM was obtained.

We then extended our investigations to eukaryotic homologs of SO0126. *D. melanogaster* and *H. sapiens* TANGO2 proteins with a Strep-tag II were expressed in *S. oneidensis* and purified (Fig. S5D). These two proteins were found to bind heme (Fig. 4E), consistent with recently published results by others (28). Intriguingly, in some TANGO2 homologs, the essential Tyr residue is replaced with Phe (Fig. 4F). To examine whether a Phe at this position also supports heme binding, SO0126^Y108F^ was constructed and expressed in the Δ*ccmI* strain. In the presence of 1 mM IPTG, the cyt *c* content was significantly elevated, albeit to a slightly reduced level compared to that observed with the WT SO0126 (Fig. 4C). Taken together with eukaryotic TANGO2 proteins, our data provided solid evidence that SO0126 is a heme-binding protein with a function in cytosolic heme trafficking (28). Based on our findings, we named SO0126 as heme-trafficking protein A (HtpA) in *S. oneidensis*.

### HtpA may deliver heme to Type I hemoproteins

**Fig 5:**
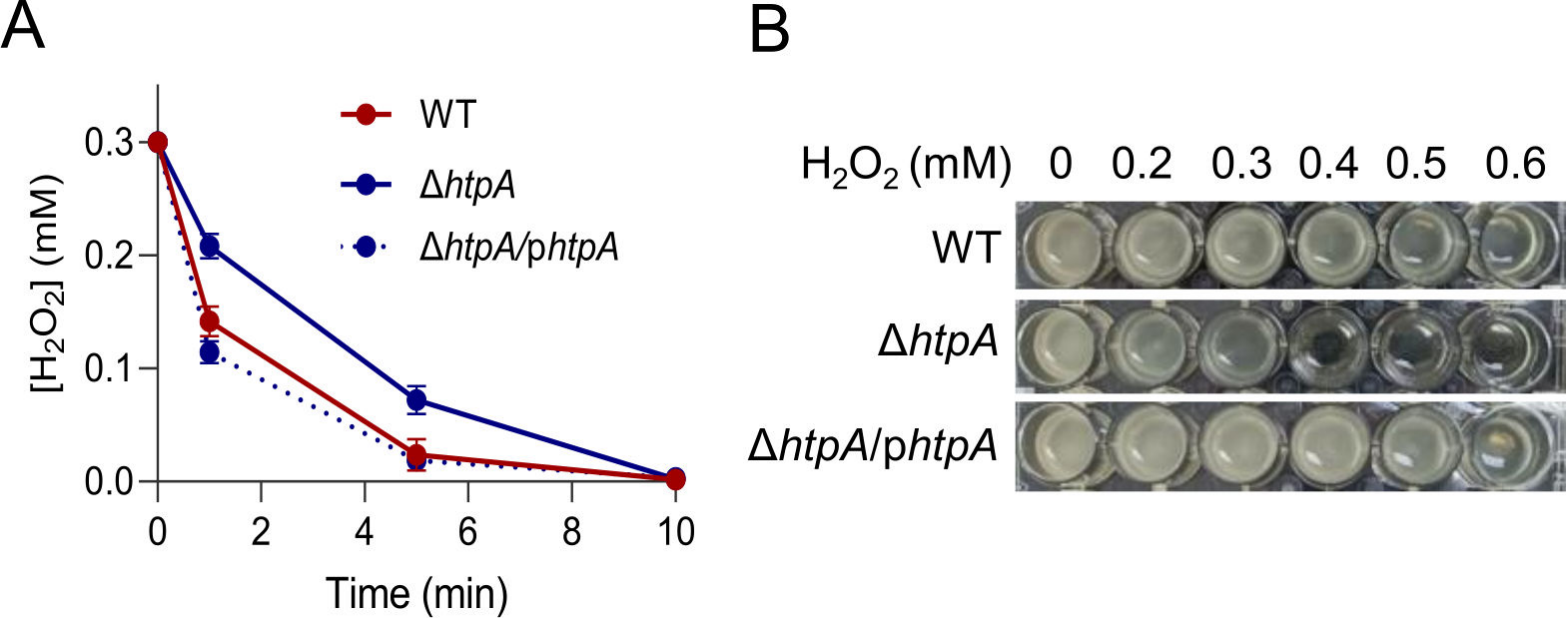
Identification of target hemoproteins of HtpA. (**A**) H_2_O_2_ consumption assay. H_2_O_2_ was added to mid-exponential phase cultures to 0.3 mM, and concentrations of the remaining H_2_O_2_ after 1, 5, 10 min were measured. The data were presented as the average of four biological replicates with error bars representing standard deviations. Complementation strain grown with IPTG at 0.2 mM was included. (**B**) H_2_O_2_ sensitivity assay. Growth of the indicated strains in 1 mL LB supplemented with H_2_O_2_ up to 0.6 mM in a 24-well plate at 30 ℃. Shown are representative results of four biological replicates after 16 h inoculation.

We then asked if HtpA delivers heme to Type I hemoproteins. We tested this notion with catalase KatB, which contains heme *b* and is the predominant enzyme for scavenging hydrogen peroxide (H_2_O_2_) in the cell (41 - 43). The H_2_O_2_ consumption assay revealed that the Δ*htpA* strain decomposed H_2_O_2_ at a rate slower than WT (Fig. 5A), and this result was confirmed by in-gel catalase staining (Fig. S6A). Consistently, the Δ*htpA* strain was found to be significantly more sensitive to H_2_O_2_ than WT; similar levels of growth inhibition by H_2_O_2_ were observed at 0.6 mM for WT and only 0.3 mM for the mutant (Fig. 5B). In addition, we tested another hemoprotein, cyt *bd*, a terminal oxidase that carries out oxygen respiration to support aerobic growth of *S. oneidensis* in the presence of 3 mM nitrite (44, 45). However, the impact of the HtpA depletion on the activity of cyt *bd* was negligible (Fig. S6B). Our data suggest that the heme-trafficking activity of HtpA may comprise assembly of some but not all Type I hemoproteins, implying that there is target specificity.

### HtpA interacts directly with the CCM apparatus

**Fig 6:**
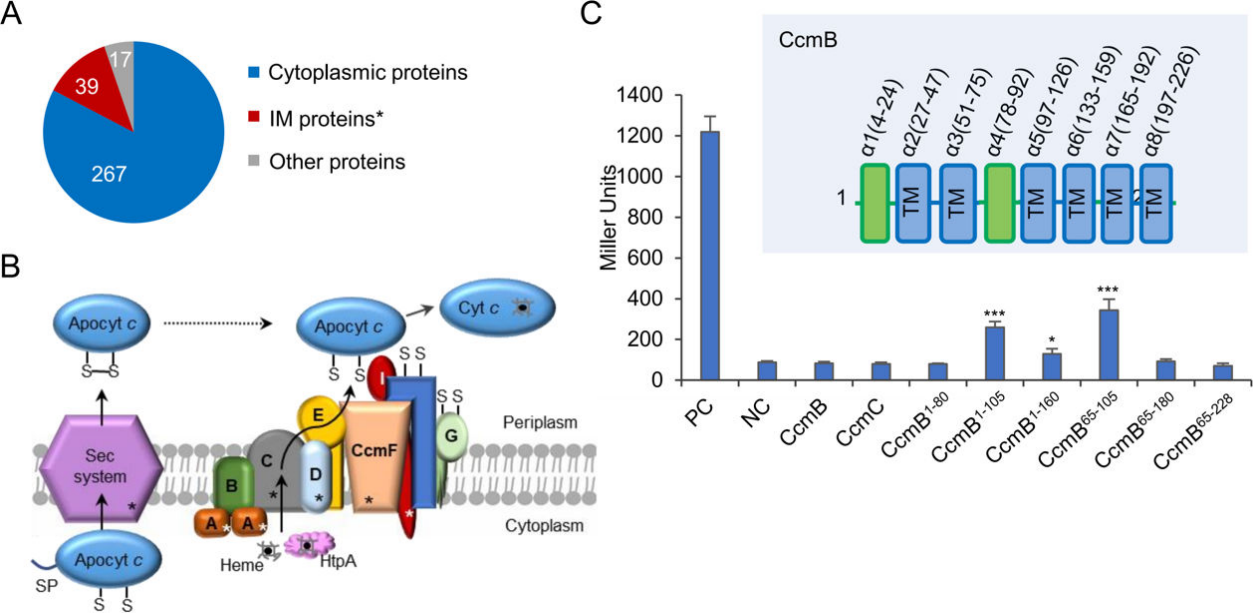
Identification of proteins that interact directly with HtpA. (**A**) Cellular distribution of biotinylated proteins detected by BioID. (**B**) Schematic for the proposed function that HtpA enhances the cytochrome *c* abundance by trafficking heme to the CCM apparatus. Biotinylated proteins are marked with an asterisk. (**C**) Bacterial two-hybrid (BATCH) assay for detecting the interaction between HtpA and its targets. The interaction activates the expression of the *lacZ* gene, which can be detected by β-galactosidase activity assay. The activities of β-galactosidase in BTH101 reporter strains carrying various combinations of vectors grown on the LB plate containing 40 µg/mL X-gal and 0.5 mM IPTG at 30°C for 24 h were measured and presented as the average of four replicates with standard deviations (error bars) in Miller Units. The secondary structural elements of CcmB are shown, in which the cytoplasmic helices are in green and TM represents transmembrane helices.

To test if HtpA delivers heme to its targets directly, we used the proximity-dependent biotin-based biochemical mapping method called BioID (46, 47). The approach employs a fusion of a bacterial biotin ligase (BirA*, an *E. coli* BirA mutant which promotes promiscuous biotinylation) to the protein of interest (the bait), which is able to biotinylate proteins in the immediate vicinity to the bait (Fig. S7A). The biotinylated proteins can be effectively enriched with streptavidin affinity matrices, allowing the identification of weak and/or transient interactions. HtpA-BirA* fusion was expressed in the *S. oneidensis* Δ*htpA* strain and was functional (Fig. S7B). A large number of proteins in this strain were found to be biotinylated in the BioID sample, in contrast to only two biotin-dependent carboxylases, LiuD (methylcrotonyl-CoA carboxylase) and SO0840 (acetyl-CoA carboxylase) (48) in the control (Fig. S7C). Biotinylated proteins were then purified and identified by liquid chromatography-tandem mass spectrometry (LC-MS/MS). MS results yielded a list of 323 candidates from the BioID fraction, with up to 80% of the non-natural biotinylated proteins reside in the cytoplasm, in line with the cytoplasmic location of HtpA (Fig. 6A; Table S1). In this list, LiuD and SO0840 were ranked as hits #3 and #5, respectively, confirming that biotinylated proteins were, indeed, enriched. Among the other top hits were four protein chaperones, including ClpB, DnaK, HtpG, and GroEL, which were readily explained by the protein-interacting nature of these proteins (49). However, KatB was not found in the hit list. The hit list of BioID sample also included 39 proteins that are either located or associated with the cytoplasmic membrane. Interestingly, five out of nine Ccm proteins were among them, including CcmA, CcmC, CcmD, CcmF, and CcmI (Fig. 6B), suggesting that HtpA may interact with the Ccm apparatus.

To confirm the interaction between HtpA and the Ccm apparatus, a bacterial two-hybrid system (BACTH), which is based on adenylate cyclase reconstitution, was employed (50). Each of the *ccm* gene was cloned into vector T18 to pair with T25-*htpA*. While strong interaction was observed in the positive control (T18-zip/T25-zip), no interacting signal was observed between any full-length Ccm component and HtpA (Fig. 6C; Fig. S8A). For CcmE, CcmF, CcmG, CcmH, and CcmI, this is not surprising since they are not directly involved in heme transport across the membrane (4, 20). In the case of heme-transporting CcmB and CcmC, we reasoned that the failure could be due to the fact that both of them are integral membrane proteins containing six transmembrane helices with only a small portion exposed to the cytoplasm (4, 20, 51). To overcome this challenge, we designed and expressed a series of CcmB and CcmC truncations within T18 to pair with T25-*htpA* (Fig. 6C; Fig. S8A). The cytoplasmic segments of both polypeptides, the α1 and α4 of CcmB or the α7 of CcmC, were included in each truncation (Fig. 6C; Fig. S8B). While none of the CcmC truncations generated positive signals, three CcmB constructs were found to interact with HtpA (Fig. 6C; Fig. S8B), substantiating the notion that CcmB is the protein that accepts heme from HtpA. Consistent with the BioID result, BACTH also failed to detect the interaction between HtpA and KatB. Altogether, these results validated that HtpA interacts with its target proteins, at least CcmB.

## DISCUSSION

Initially predicted to play roles in secretory protein loading in the endoplasmic reticulum, TANGO2 proteins of multiple eukaryotic organisms were recently demonstrated to be a heme-binding protein trafficking heme from the surface of the mitochondria to hemoproteins within the cytosol (27, 28). In this study, using a high-throughput approach to screen for proteins impacting the cyt *c* content in *S. oneidensis*, we identified HtpA as a bacterial homolog of TANGO2, which in overproduction promotes cyt *c* biosynthesis. A relatively comprehensive genetic and biochemical analysis uncovered that this protein is a heme-binding protein, which may be involved in the assembly of multiple hemoproteins, including cyts *c* and catalase. Intriguingly, HtpA/TANGO2 homologs are widespread across all domains of living organisms, but because of parallel evolution, these proteins are largely non-exchangeable in physiological roles. Thus, our findings confirm and extend available data on TANGO2 proteins.

*S. oneidensis* is characterized by two distinct characteristics with respect to cyt *c*, the exceptional abundance of cyt *c* and the non-essential role of CcmI in cyt *c* biosynthesis (23, 29). These features enabled us to develop a high-throughput screening for proteins that significantly increase the cyt *c* content. Our approach was validated by the observation that proteins known to catalyze biosynthesis and degradation of cAMP, which is closely linked to the cyt *c* content (31), were among the mutants identified. In addition, we discovered HtpA, which was an uncharacterized protein in *S. oneidensis* prior to this study. The homologs of HtpA are widespread, not only present in three domains of cellular organisms but also present in a few viruses. Notably, these proteins are missing in the order of enterobacteriales, which contain some of the best-studied model organisms, such as *Escherichia* and *Salmonella*. On the other hand, the HtpA homologs are omnipresent in eukaryotes where they are named TANGO2 (27). Nonetheless, a statistically confident evolutionary relationship among HtpA/TANGO2 was difficult to define, even for those from organisms within the same family. This suggests that these proteins were largely subjected to parallel molecular evolution. As a result, most of bacterial homologs were unable to functionally replace HtpA in *S. oneidensis*, let alone TANGO2 proteins. In fact, functional complementation for HtpA could only be achieved by its closest cousins in the phylogenetic tree. We speculate that a common ancestor possessing the heme-binding trait gave rise to all modern HtpA/TANGO2 proteins. While the modern proteins still are able to bind heme, they have been honed by evolution to adapt to their respective habitats, resulting in mechanistic differences that are sufficiently significant to amount to non-exchangeability.

HtpA binds to heme with a 1:1 stoichiometry and a *K*_d_ of 1.2 × 10^−5^ M, implying that the binding is not very tight. In a recent report, the TANGO2 proteins of mice, yeast, and human were demonstrated to function as a heme-trafficking protein with a rather low *K*_d_ (1.54 × 10^−5^ M, yeast TANGO2) (28). The heme-binding affinities of TANGO2 and HtpA are in the same range, similar to those of bacterial hemoproteins suggested to be able to traffick heme, such as *E. coli* ChuX (*K*_d_ = 1.99 × 10^−5^ M), but substantially different from hemophore proteins, such as HasA of *P. aeruginosa* which has a very high affinity for heme (*K*_d_ = 1.8 × 10^−11^ M) (52 - 54). Additionally, by using the alanine scanning, we validated that residue Tyr^108^ of HtpA is required for heme binding, in line with that Tyr functions as heme ligand in some proteins (40). Because Tyr^108^ is replaced by Phe in some homologs (i.e., *S. cerevisiae*), we also showed that the HtpA^Y108F^ variant could partially complement the HtpA loss. This result not only indicates that the tyrosine is not absolutely essential for heme-binding but also confirms heme-binding capacity of *S. cerevisiae* TANGO2 (28). Given that phenylalanine and tyrosine have similar side chains, we assume that the benzene ring may be important for the heme coordination.

By employing the BioID screening and BACTH, we provided evidence to substantiate HtpA as a heme-trafficking protein for the CCM apparatus. The BioID screening revealed that the majority of the CCM components were labeled by biotin, presumably due to their location proximity to HtpA. Among them, CcmB was validated by BACTH to be the only CCM component that interacts with HtpA directly. In addition to six TM helices, CcmB contains two additional helices, α1 and α4, located in the cytoplasm (4, 20, 51) (Fig. 6C). It appears that α4 is essential for the interaction. BACTH failed to detect an interaction between HtpA and CcmC although CcmC resembles CcmB in structure (Fig. S8B) (20). The possibility that HtpA may also interact with other CCM components cannot be completely eliminated as the interaction between HtpA and full-length CcmB was also elusive from the BACTH analysis. We envision that this is probably due to the topology of CcmB, which only has a small portion exposed to the cytoplasm (20). The majority of CCM components are topologically alike, which likely poses a similar issue for BACTH to work with these proteins.

Although HtpA in overproduction substantially elevated the cyt *c* content, its absence resulted in a modest down-regulating effect. Potential reasons for this observation include that multiple heme-trafficking proteins contribute to delivering heme to the CCM apparatus, that heme is mobilized independent of any heme-trafficking proteins, and that the aforementioned mechanisms work in combination. In addition to the cyt *c* content, the activity of catalase KatB, which is a Type I hemoprotein dictating H_2_O_2_ degradation in *S. oneidensis* (43), was also subjected to regulation of HtpA. Our findings are in line with the proposal that the assembly of catalase *in vivo* is assisted by chaperones (55), albeit such proteins had not been identified. Nevertheless, neither BioID nor BACTH detected an interaction between HtpA and KatB, coinciding with a previous report that heme-trafficking protein HemW of *E. coli* was found to interact with two hemoproteins but not catalase (16). Therefore, all available evidence points to a scenario where heme trafficking proteins such as HtpA/TANGO2 make weak and often highly transient interaction with their protein targets, consistent with their expected role to deliver heme to a large array of diverse hemoproteins. Future biochemical and structural studies using recombinant proteins are needed to reveal further molecular details of such interactions and the working mechanisms of HtpA/TANGO2.

## MATERIALS AND METHODS

### Bacterial strains, plasmids, and culture conditions

The bacterial strains and plasmids used in this study are listed in Table S2A. The sequences of the primers used in this study are listed in Table S2B. All the chemicals were obtained from Sigma-Aldrich (Shanghai, China) unless otherwise noted. The restriction endonucleases and T4-ligase were obtained from Thermofisher (Shanghai, China). *E. coli* and *S. oneidensis* were grown in Lennox LB (Difco, Detroit, MI, USA) under aerobic conditions at 37°C and 30°C for genetic manipulation. When appropriate, the growth medium was supplemented with the following: 2,6-daminopimelic acid (DAP) at 0.3 mM; ampicillin at 100 µg/mL; kanamycin at 50 µg/mL; gentamicin at 15 µg/mL; d-biotin at 15 mg/L.

For physiological characterization, *S. oneidensi*s strains were cultured in Lennox LB or defined minimal medium (MM) as described previously (56). Similar results were obtained with the two media, and therefore, only the data from using Lennox LB were presented. Growth was monitored by measuring the optical density at 600 nm (OD_600_). For aerobic growth, 3 mL fresh medium was inoculated with 30 µL overnight culture and incubated on a shaker (250 rpm) at 30°C. For anaerobic growth, the overnight culture grown aerobically was purged with nitrogen gas and inoculated to ~0.01 (OD_600_) in fresh medium prepared anaerobically. TMAO (20 mM) was used as the electron acceptor in this study to support anaerobic growth.

### Mutagenesis and controlled expression for complementation

In-frame deletion strains of *S. oneidensis* were constructed according to the *att*-based Fusion PCR method as described previously (29). In brief, two fragments flanking the target gene were amplified by PCR with primers containing *attB* and the gene-specific sequence and then fused by a second round of PCR. The fusion products were introduced into plasmid pHGM01 using the Gateway BP Clonase II enzyme mix (Invitrogen, Shanghai, China) according to the manufacturer’s instructions. The resulting vectors were maintained in *E. coli* DAP auxotroph WM3064 and subsequently transferred into relevant *S. oneidensis* strains via conjugation. For conjugation, 0.2 mL of *E. coli* donor cells and 0.8 mL of *S. oneidensis* receiver cells, both of which were grown to late-exponential phase in Lennox LB, were mixed and placed on a Lennox LB plate supplemented with required chemicals, and the plate was incubated at 30°C overnight. Integration of the deletion constructs into the chromosome was selected by resistance to gentamicin and confirmed by PCR. Verified transconjugants were grown in Lennox LB in the absence of NaCl and plated on Lennox LB supplemented with 10% sucrose. Gentamicin-sensitive and sucrose-resistant colonies were screened by PCR for the intended deletions. Mutants were verified by sequencing the mutated region.

Genetic complementation of all mutants used in this study was carried out with IPTG-inducible gene expression vector pHGEN-P*tac* (57). The gene of interest was generated by PCR and introduced into the vector after promoter P*tac*. After verification by sequencing, the resultant vectors were transferred into the relevant strains via conjugation. Expression of the gene of interest was induced by IPTG.

### Transposon screening

Mariner-based transposon vector pHGT01 was transferred from *E. coli* WM3064 into *S. oneidensis* Δ*ccmI* via conjugation (30). The bacterial mixture on the conjugation plate was washed off with 10 mL fresh Lennox LB, and the resulting cell suspension was divided into aliquots, each of which was vortexed gently for cell separation and then incubated on a shaker (200 rpm) for 30 min. Two hundred microliters of cell suspension was spread onto the selective plates containing gentamicin. The plates were incubated for 36 h at 30°C. Colonies with more intensified red color were selected and subjected to transposon insertion mapping with arbitrarily primed PCR (58).

### GFP fusion visualization

To determine the cellular location of HtpA, DNA fragments encoding HtpA-GFP fusion proteins with or without the PetA signal peptide sequence were synthesized. The resulting DNA fragments were cloned into pHGE-P*tac* and transferred to the *S. oneidensis* WT strain. Cells expressing the GFP fusion proteins were grown to the mid-exponential phase and visualized with LSM710nlo laser confocal microscope (Carl Zeiss, Germany) as described previously (35).

### Cyt *c* and heme *b* assays

The cell color analysis, used to conveniently estimate the cyt *c* abundance, was carried out with colonies on Lennox LB plates and cell pellets from cultures grown in Lennox LB to the early stationary phase. Cultures containing similar numbers of cells were centrifuged, and pellets were photographed and subsequently subjected to heme quantification. Total heme was measured per micrograms of protein using either the QuantiChrom heme assay kit (BioAssay Systems, Hayward, CA, USA) or the Turbo-TMB (3,3′,5,5′-tetramethylbenzidine) assay if the kit is not available (59). Sample preparation for both methods is the same. The pellets were washed twice with cold phosphate buffer saline (PBS, 137 mM NaCl, 2.7 mM KCl, 10 mM Na_2_HPO_4_, 1.8 mM KH_2_PO_4_, pH 7.4) and suspended in PBS to an OD_600_ of ~0.5. An aliquot of 100 µL suspended cells was lysed, and 25 µL resultant samples were transferred wells of a 96-well plate for the reaction and measurement. For the kit, the addition of reagent and measurement were carried out according to the manufacturer’s instructions. For the Turbo-TMB assay, a volume of 100 µL Turbo-TMB solution was added to the well, mixed by tapping the plate, and then incubated for 30 min at room temperature (59). The reaction was stopped by adding 2 M sulfuric acid (125 µL), and the absorbance of the reaction mixture at 450 nm was measured using TECAN infinite M200PRO (Mannedorf, Swiss). Heme solutions (final concentrations ranged from 4 to 150 nM) were used to generate the standard. To quantify the cyt *c* content, the whole bacterial proteome was extracted by using Bacterial Protein Extraction Kit (Sangon Biotech, Shanghai, China). Throughout this study, the protein concentration of the resulting cell lysates was determined using a Bradford assay with bovine serum albumin as a standard (Bio-Rad) or using a GE NanoVue Spectrophotometer for fast assessment. The extracted proteome was subjected to denaturation by trichloroacetic acid (TCA) (8%, final concentration) precipitation to release non-covalently attached heme, and the precipitated protein portion was used for heme *c* measurement with the QuantiChrom heme assay kit or the Turbo-TMB assay. The free heme content was the difference in concentrations of total heme and heme *c*.

### Site-directed mutagenesis

Site-directed mutagenesis was performed using a QuikChange II XL site-directed mutagenesis kit (Agilent, Beijing, China) (60). The *htpA* gene within pHGE-P*tac*-SO0126 was subjected to modification, and the resulting products were digested by *Dpn*I at 37°C for 6 h and subsequently transformed into *E. coli* WM3064. The vectors were verified by sequencing and transferred into the *S. oneidensis* strains by conjugation.

### Susceptibility assay

Susceptibility of cells to H_2_O_2_ and nitrite (NaNO_2_) was evaluated by multiple approaches. Impacts of H_2_O_2_ on cell growth were assessed in LB supplemented with H_2_O_2_ up to 0.6 mM in 24-well plates. The plates were incubated at 30°C, and photos were taken 16 h later. Inhibitory effects of nitrite on cell growth were examined on LB plates supplemented with nitrite up to 5 mM. Cultures of the mid-exponential phase (OD_600_ ≈ 0.4) were adjusted to approximately 10^8^ CFU/mL (dilution factor, 0), followed by 10-fold serial dilution. A 5 µL volume of each dilution was spotted onto LB plates containing nitrite up to 5 mM, which were incubated at 30°C for at least 24 h before being photographed.

### Analysis of catalase

Catalase activity was determined by H_2_O_2_ decomposition assay and catalase staining as described before (45). In H_2_O_2_ decomposition assay, cultures of the mid-exponential phase were aliquoted and H_2_O_2_ of different concentrations was added into each aliquot. One, five, and ten minutes after the addition, aliquots were assayed for the remaining H_2_O_2_ by using the ferrous ion oxidation-xylenol orange (FOX) reagent (61). In catalase staining, cells grown to the mid-exponential phase were collected after a 10-min H_2_O_2_ treatment (0.2 mM), and proteins of cells lysates were separated by 10% nondenaturing polyacrylamide gel electrophoresis (PAGE) and stained with ferricyanide (62).

### BATCH

BATCH was used to test the direct interaction between HtpA and its potential targets (50). The *htpA* gene and each of the target genes were cloned into pKT25 and pUT18, respectively, and the resulting vector pair was co-transformed into *E. coli* BTH101. The strains having the vector pair were selected on LB plates containing 100 µg/mL ampicillin, 50 µg/mL kanamycin, 40 µg/mL X-gal, and 0.5 mM IPTG. β-Galactosidase activity was determined on a TECAN infinite M200PRO (63).

### Analysis of promoter activity

The activity of promoters of interest was assessed using a single-copy integrative *lacZ* reporter system as described previously (64). Briefly, DNA fragments containing the sequence upstream of the target operon (up to 500 bp relative to the translation start codon) were generated by PCR and cloned into reporter vector pHGI01. After conjugated into *S. oneidensis*, the resulting vectors integrated into the chromosome and the antibiotic marker was then removed by an established approach (46). Cells grown to the mid-exponential phase under specific conditions were collected, and β-galactosidase activity was determined.

### Purification of Strep-tag II recombinant and biotinylated proteins

Recombinant HtpA, HtpA^Y108A^, *Hs*TANGO2, and *Dm*TANGO2 with Strep-tag II were produced in the *S. oneidensis* Δ*htpA* strain. After induction by 1 mM IPTG for 16 h at 16°C, cells from 2 L cultures were harvested at 2,500 g for 20 min, washed twice with cold Tris-HCl binding buffer (20 mM Tris-HCl, 150 mM NaCl, 1 mM EDTA, pH 8.0), and resuspended in 40 mL of the same Tris-HCl buffer. After the addition of 1 mM phenylmethanesulfonyl fluoride (PSMF) and 50 µg/mL DNase I, the resuspended cells were disrupted at 1,000 bar for four times in a JN-10C French Press (JNBIO, Guangzhou, China), and the protein extract (the supernatant) was obtained by centrifugation at 25,000 *g* for 30 min. The extract was inoculated with Beads Magrose Strep-Tactin (Solarbio, Beijing, China) at 4°C for 1 h, and the protein bound to Beads was eluted by 1–2 mL elution buffer (20 mM Tris-HCL, 150 mM NaCl, 1 mM EDTA, 2.5 mM dethiobiotin, pH 8.0). The purified protein was stored in an elution buffer and diluted with a binding buffer in the follow-up experiments.

### SDS-PAGE, Western blotting, and heme staining

Cells grown in LB to the mid-exponential phase before and after induction of IPTG at proper concentrations for 10 h were collected and lysed by sonication with a Xinzhi Sonifier (JY92-IIDN, NingBo, China) at maximum output at 0°C until no whole bacterial cells were visible, and the protein extracts were collected after centrifugation at 3,000 *g* for 10 min. The extracts were mixed with 4× SDS-loading buffer, denatured for 10 min at 100°C, and resolved by 12% SDS-PAGE gel. The resolved proteins were directly stained either with Coomassie Brilliant Blue R-250 or with 3,3′,5,5′-tetramethylbenzidine for heme-staining analysis (65) or subject to Western blotting. Protein transfer onto the PVDF membrane (GE-Healthcare, Shanghai, China) was carried out for 1 h at 60 V in a Criterion blotter (Bio-Rad, Shanghai, China) with Tris-Glycine transfer buffer. To detect proteins with Strep-tag II, the blotting membrane was probed with the primary antibody mouse anti-Strep-tag II monoclonal antibody (1:5,000) (Abbkine, Shanghai, China) and then the second antibody goat anti-mouse IgG-HRP (1:100,000) (Beyotime, Beijing, China). To detect biotinylated proteins, HPR-streptavidin was used as the probe. The blots were finally developed by chemiluminescence detection with SuperSignal West Dura Extended Duration Substrate kit (Invitrogen, Shanghai, China) and visualized with the CLiNX system.

### Heme binding and affinity assays

Hemin chloride was dissolved in DMSO prior to each experiment. The binding assay was carried out with hemin of different concentrations and 10 µM protein in the binding buffer (150 mM NaCl, 50 mM Tris-HCl, pH 7.9) w/o 5 mM sodium dithionite (DTT) at 20°C for up to 6 h in the dark. Absorption spectra were recorded from 260 to 600 nm for 200 µL samples using UV-visible spectroscopy (TECAN infinite M200PRO). Changes in the Soret peak (A_412_ for hemoprotein) were plotted as a function of hemin concentration.

### Tryptophan fluorescence quenching assay

The dissociation constant (*K*_d_) of HtpA and heme was determined by monitoring the quenching of intrinsic fluorescence from the single tryptophan residue of HtpA upon heme binding (66). Hemin at concentrations up to 20 µM w/o 10 mM DTT was added to 200 µL sample containing 2, 5, and 10 µM protein with 25 µM NATA [4,4′4″-Tris (*N*,*N*-diphenylamino) triphenylamine]. The fluorescence was excited at 285 nm and recorded between 330 and 500 nm. Spectra were measured in triplicate, and the experiment was repeated three times. The dissociation constant *K*_d_ was calculated using the equation (*F*_0_ *− F*)/(*F − F*_max_) = ([heme]/*K*_d_)^*n*^, where *F* represents fluorescence intensity in the area under the fluorescence spectra (330–500 nm), *F*_0_ represents the fluorescence intensity of protein alone, and *F*_max_ represents the fluorescence intensity of protein saturated with heme, while *n* represents the number of equivalent binding sites (the stoichiometry), which was 1.

### Proximity-dependent biotin-based biochemical mapping

BioID was performed with Δ*htpA* expressing the HtpA-BirA^*^ fusion following an established protocol (47). The BirA^*^ used here was derived from the BirA of *S. oneidensis* after site-directed mutagenesis. A DNA fragment for the HtpA-BirA^*^ fusion was generated by fusion PCR and cloned into vector pHGE-P*tac*, resulting in pHGE-P*tac*-HtpA-BirA^*^, which was transferred into Δ*htpA* after sequencing verification. The strain was grown in 2 L LB supplemented with 50 µg/mL kanamycin at 200 rpm at 30°C to the mid-exponential phase, and then 15 µg/mL d-biotin and 0.2 mM IPTG were added to induce expression of HtpA-BirA^*^ for 16 h. The cells were collected at 2,500 g for 20 min, and the cell pellets were washed twice with cold PBS (137 mM NaCl, 2.7 mM KCl, 10 mM Na_2_HPO_4_, 1.8 mM KH_2_PO_4_, pH 7.4) and resuspended in PBS. The soluble extract was obtained from the pellets by using B-PER Direct Bacterial Protein Exaction Kit (ThermoFisher, Shanghai, China) . The extracts were inoculated with Biotin immunomagnetic beads (BBI, Shanghai, China) at 4°C for 1 h, and the protein was eluted by 1–2 mL elution buffer (8 M Guanidine·HCl, pH 1.5). The elution sample was analyzed by LC-MS/MS (Applied Protein Technology, Shanghai, China) for the identification of the biotinylated proteins.

### *In silico* analysis and statistics

HtpA homologs with different BLASTp *E*-values as cutoff were identified by BLASTp and HMMER (37). Distribution of the homologs and similarity comparison of HtpA and *Hs*TANGO2 were analyzed with HMMER. The Bootstrap consensus trees were constructed by using MEGA-X by the neighbor-joining statistical method with Poisson model as the substitution model and 1,000 of Bootstrap replications (67). The topology of HtpA was predicted with TPOCONS (34). The predicted structure of *Hs*TANGO2 was obtained from AlphaFold Protein Structure Database (38), and the structure of HtpA was predicted by AlphaFold2. The structure superimposition was operated on PyMOL (www.pymol.org). Sequence alignments were generated with ClusterIX and Espript (68). Statistical significance was calculated by using Student’s *t*-test for pairwise comparisons and using one-way ANOVA with the Student–Newman–Keuls multiple comparison test in GraphPad Prism 8.4.2 (GraphPad, San Diego, CA, USA). Data values were presented as mean ± SEM. A *P* value < 0.05 was considered significant (69, 70).

## Data Availability

All the data associated with this work are included either in the paper or in the online supplemental materials.

## SUPPLEMENTAL MATERIAL

Fig. S1 mbio.01320-23-s0001.pdf Characteristics of SO0126 and SO0127 in *Shewanella*.

Fig. S2 mbio.01320-23-s0002.pdf Topology and structure of SO0126 and its homolog.

Fig. S3 mbio.01320-23-s0003.pdf Phylogenetic analysis of bacterial homologues of SO0126.

Fig. S4 mbio.01320-23-s0004.pdf SO0126 has no significant effect on heme levels.

Fig. S5 mbio.01320-23-s0005.pdf SO0126 and TANGO2 are heme-binding proteins.

Fig. S6 mbio.01320-23-s0006.pdf Identification of downstream hemoproteins of HtpA.

Fig. S7 mbio.01320-23-s0007.pdf Validation of BioID.

Fig. S8 mbio.01320-23-s0008.pdf Identification of proteins interact directly with HtpA.

Table S1 mbio.01320-23-s0009.pdf Proteins identified by BioID with HtpA as the bait in *S. oneidensis*.

Table S2 mbio.01320-23-s0010.pdf Strains, plasmids, primers used in this study.

ASM does not own the copyrights to Supplemental Material that may be linked to, or accessed through, an article. The authors have granted ASM a non-exclusive, world-wide license to publish the Supplemental Material files. Please contact the corresponding author directly for reuse.
