# Supplementary material for: Bacterial TANGO2 homologs are heme-trafficking proteins that facilitate biosynthesis of cytochromes c
Source: mBio. 2023 Jul 18;14(4):e01320-23. doi: 10.1128/mbio.01320-23 (PMC10470608; doi:10.1128/mbio.01320-23)
Supplement: Fig. S1 — Characteristics of SO0126 and SO0127 in Shewanella. [file mbio.01320-23-s0001.pdf]

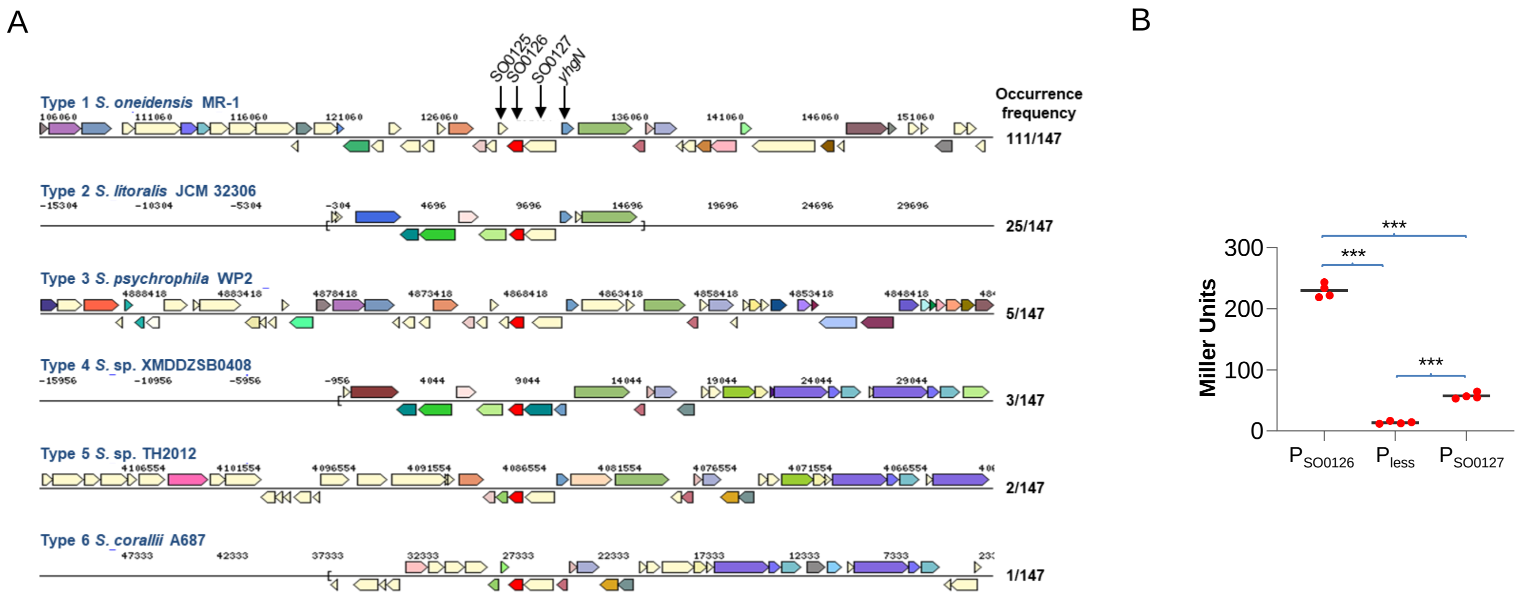

FIG S1. Characteristics of SO0126 and SO0127 in *Shewanella*. (A) Perfect conservation in the distribution and the gene arrangement of SO0126 and SO0127 in *Shewanella*. All *Shewanella* strains whose genome sequences are available were analyzed. Based on the genes flanking the SO0126-SO0127 locus, 6 different types exist, with Type 1, as in *S. oneidensis*, being prevailing. Nevertheless, SO0126-SO0127 are perfectly conserved. Note that in Type 4, the counterpart of SO0127 is in different color but the BLASTp E-value between these two proteins is 9e-168, indicating that they are highly homologous. (B) Promoter activity assay. The promoter regions for SO0126 and SO0127 were cloned to integrative *lacZ* reporter vector and introduced into WT for chromosome integration. P_{less}, a promoter-less DNA fragment as the negative control. After verification, cells carrying the reporter systems grown to the mid-exponential phase were collected for β-galactosidase activity assay, presented in Miller Units. Throughout this study, shown are the data from biological replicates with asterisks indicating statistically significant difference of the values compared ($n = 4$; ns, not significant; *, $p < 0.05$; **, $p < 0.01$; ***, $p < 0.001$) unless otherwise noted.
