# Supplementary material for: Bacterial TANGO2 homologs are heme-trafficking proteins that facilitate biosynthesis of cytochromes c
Source: mBio. 2023 Jul 18;14(4):e01320-23. doi: 10.1128/mbio.01320-23 (PMC10470608; doi:10.1128/mbio.01320-23)
Supplement: Fig. S2 — Topology and structure of SO0126 and its homolog. [file mbio.01320-23-s0002.pdf]

A

B

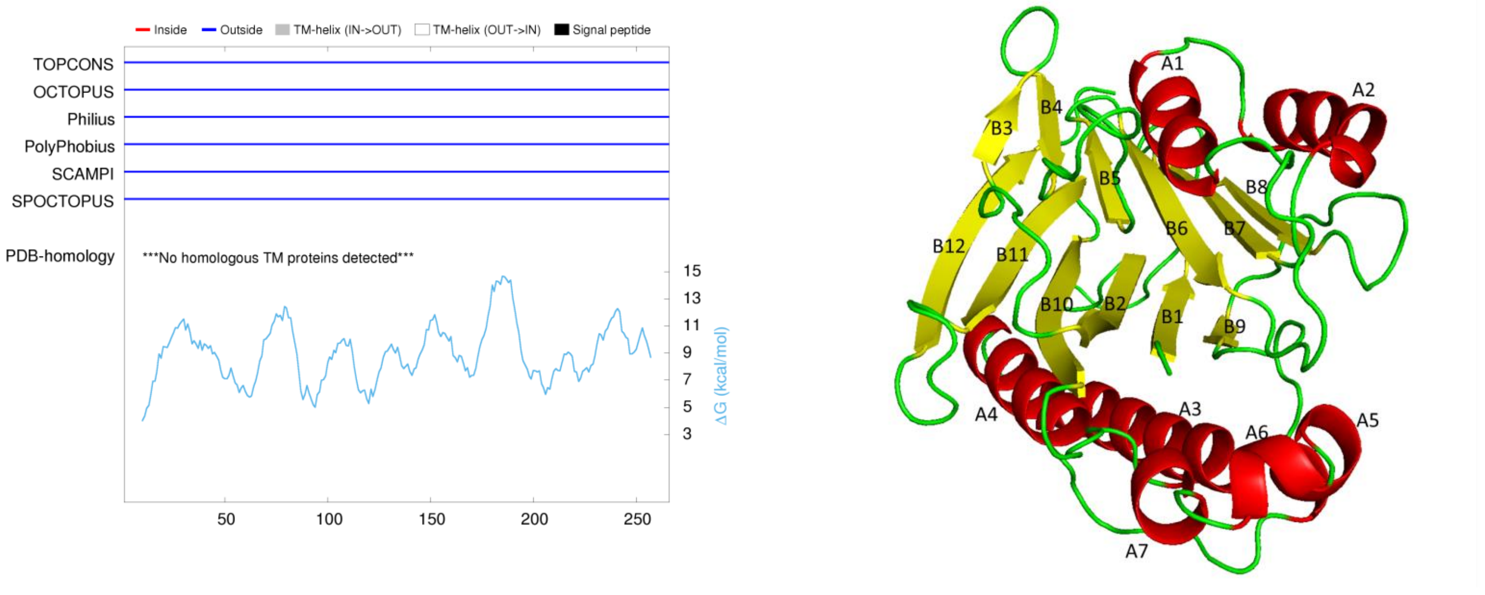

FIG S2. Topology of SO0126 predicted with TPOCONS (A) and predicted structure of *H. sapiens* TANGO2 (B) available in AlphaFold Protein Structure Database. α-helices and β-strands represented by A and B respectively, are numbered from the N-terminus.
