# Supplementary material for: Bacterial TANGO2 homologs are heme-trafficking proteins that facilitate biosynthesis of cytochromes c
Source: mBio. 2023 Jul 18;14(4):e01320-23. doi: 10.1128/mbio.01320-23 (PMC10470608; doi:10.1128/mbio.01320-23)
Supplement: Fig. S3 — Phylogenetic analysis of bacterial homologues of SO0126. [file mbio.01320-23-s0003.pdf]

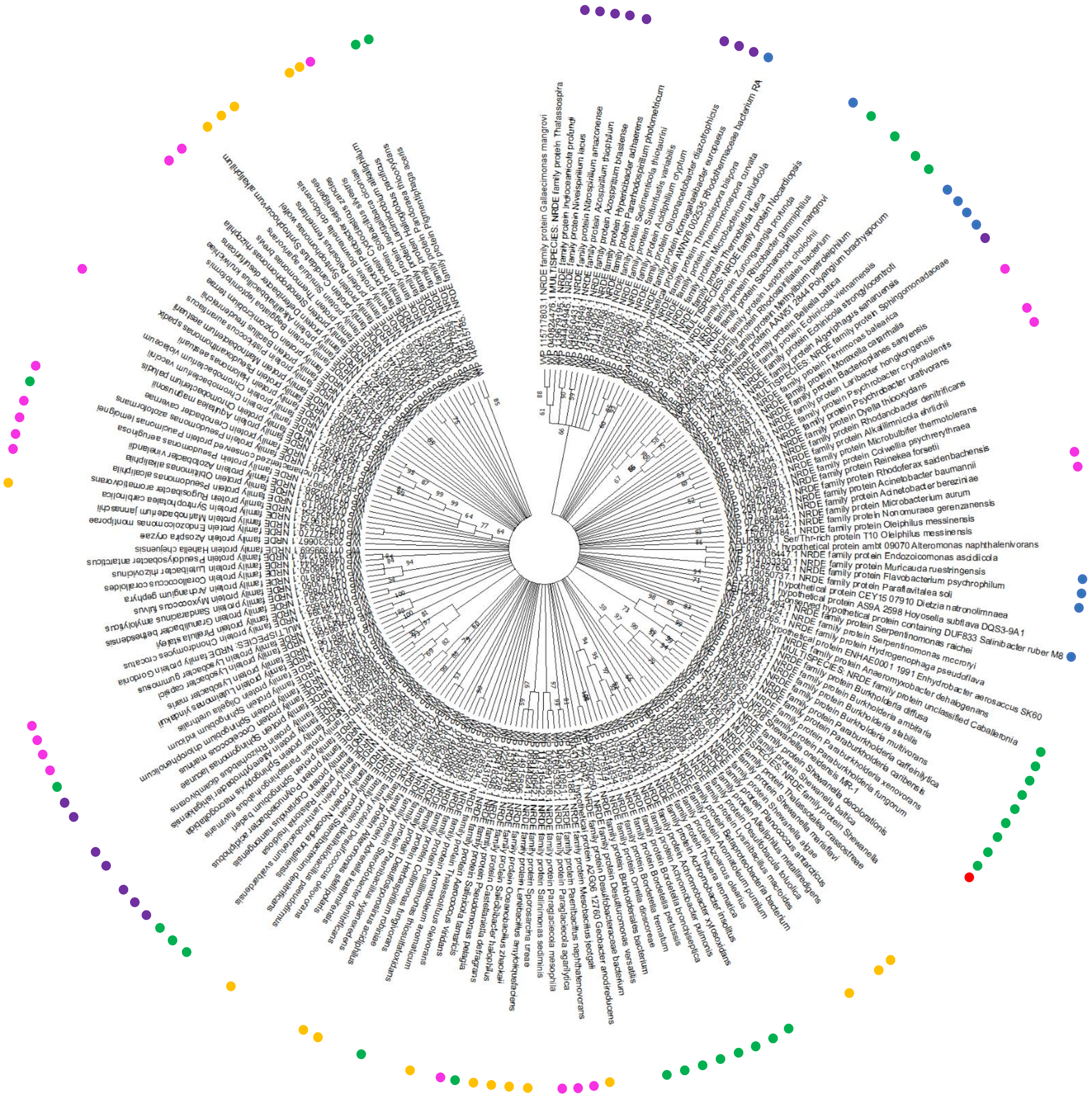

FIG S3. Phylogenetic analysis of bacterial homologs of SO0126. The Bootstrap consensus tree with 50% cutoff was constructed by the methods described in the Materials and Methods. Red dot, SO0126; purple dots, *Acetobacteraceae*, *Sphingomonadaceae* and *Azospirillaceae*; green dots, *Burkholderiaceae* and *Alcaligenaceae*; pink dots, *Moraxellaceae*, *Alteromonadaceae*, *Pseudomonadaceae* and *Xanthomonadaceae*; blue dots, Cytophaga-Flexibacter-Bacteroides group strains; and yellow dots, *Firmicutes*.
