# Supplementary material for: Bacterial TANGO2 homologs are heme-trafficking proteins that facilitate biosynthesis of cytochromes c
Source: mBio. 2023 Jul 18;14(4):e01320-23. doi: 10.1128/mbio.01320-23 (PMC10470608; doi:10.1128/mbio.01320-23)
Supplement: Fig. S5 — SO0126 and TANGO2 are heme-binding proteins. [file mbio.01320-23-s0005.pdf]

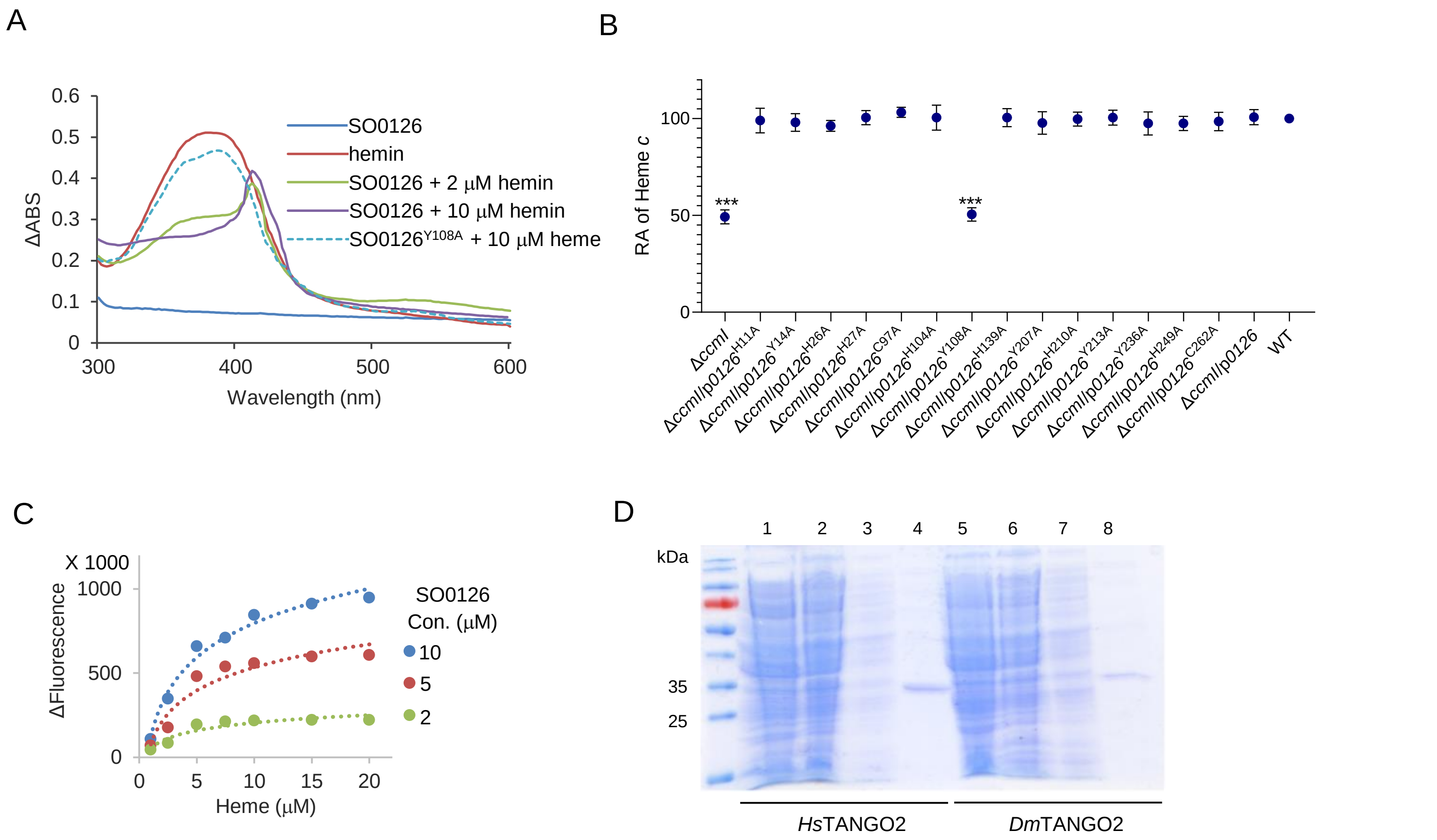

FIG S5. SO0126 and TANGO2 are heme-binding proteins. (A) Subtractive UV-visible spectra of SO0126 and SO0126^{Y108A}. In the cuvette, 10 μM purified SO0126, 2 or 10 μM hemin, without or with 5 mM DTT (representing heme) was incubated in the dark at 20 ºC up to 6 h and the absorption spectra from 300 to 600 nm were recorded. (B) The cyt *c* content in Δ*ccmI* overexpressing SO0126 variants. The data were presented as the average of four biological replicates with error bars representing standard deviations. (C) Tryptophan fluorescence quenching assay. To determine the dissociation constant (K_d) of SO0126 and heme, up to 50 μM hemin were added into 2, 5, 10 μM protein solution with 25 μM NATA. The excitation wavelength was 285 nm and the emission wavelength was from 330 to 500 nm. K_d was calculated with the equation $(F_0\text{-}F)/(F\text{-}F_{max}) = ([\text{heme}]/K_d)^n$. (D) Purification of of *H. sapiens* and *D. melanogaster* TANGO2 proteins carrying Strep-tag II. Lane 1 and 5, cell extracts before purification; lane 2 and 6. flow-through; lane 3 and 7. wash-out; lane 4 and 8, elution.
