# Supplementary material for: Bacterial TANGO2 homologs are heme-trafficking proteins that facilitate biosynthesis of cytochromes c
Source: mBio. 2023 Jul 18;14(4):e01320-23. doi: 10.1128/mbio.01320-23 (PMC10470608; doi:10.1128/mbio.01320-23)
Supplement: Fig. S6 — Identification of downstream hemoproteins of HtpA. [file mbio.01320-23-s0006.pdf]

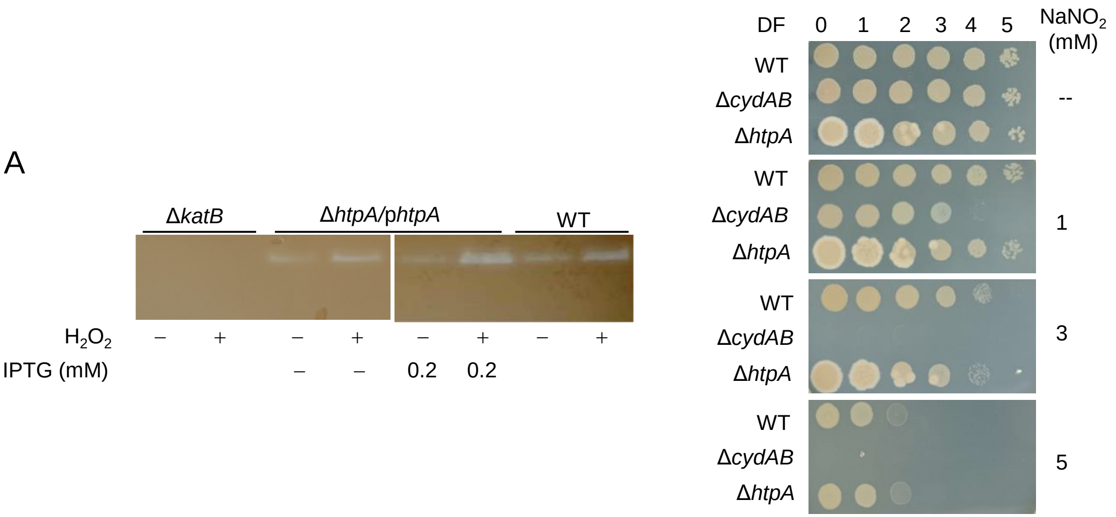

FIG S6. Identification of target hemoproteins of HtpA. (A) Catalase staining. Cells were collected before and after 0.2 mM H_2O_2 treatment for 10 min. Proteins of indicated cells lysates were separated by native PAGE and stained with ferricyanide. Catalase-free mutant (Δ*katB*) was used as the negative control. Shown are representative results of three biological replicates. (B) Nitrite susceptibility assay. Cultures of the mid-exponential phase of indicated strains were adjusted to similar optical densities (DF, dilution factor; 0 represents undiluted culture, having a cell density of approximately 10^8 cfu/ml) and gone through 10-fold serial dilution. Five microliter of each dilution was dropped onto LB plates containing varying concentrations of nitrite. Δ*cydAB*, the strain devoid of cyt *bd* oxidase. The plates were incubated at 30 °C and photos were taken 16 h later. Shown are representative results of three biological replicates.
