# Supplementary material for: Bacterial TANGO2 homologs are heme-trafficking proteins that facilitate biosynthesis of cytochromes c
Source: mBio. 2023 Jul 18;14(4):e01320-23. doi: 10.1128/mbio.01320-23 (PMC10470608; doi:10.1128/mbio.01320-23)
Supplement: Fig. S7 — Validation of BioID. [file mbio.01320-23-s0007.pdf]

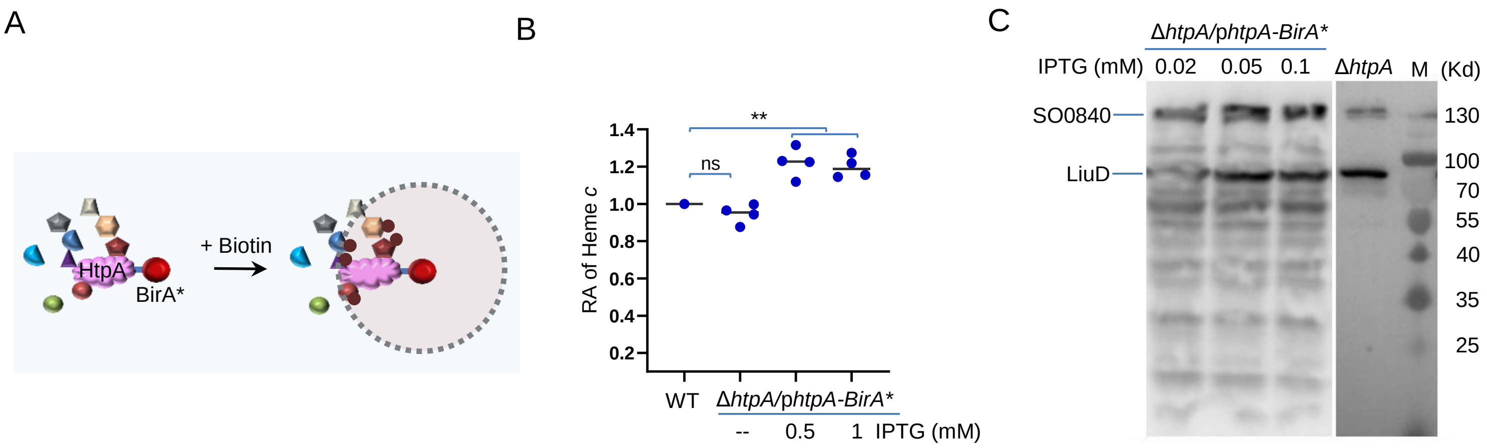

FIG S7. Validation of BioID. (A) Working Schematic for BioID. Proximity biotinylation by the bacterial biotin ligase BirA* fused to the C-terminus of HtpA. BirA* catalyzes the reaction that biotinylates the primary amines of proximal proteins within 10 nm radius. Labeled proteins are marked with dark-red dots. (B) The cyt *c* contents of strains expressing HtpA-BirA* fusion protein. (C) Biotinylated proteins in Δ*htpA/phtpA-BirA**. Shown are Western blot results conducted with the cells of indicated strains that were collected from 2 ml culture after 16 h induction with IPTG and 15 mg/L biotin, and HPR-streptavidin was used as the probe.
