# Supplementary material for: Bacterial TANGO2 homologs are heme-trafficking proteins that facilitate biosynthesis of cytochromes c
Source: mBio. 2023 Jul 18;14(4):e01320-23. doi: 10.1128/mbio.01320-23 (PMC10470608; doi:10.1128/mbio.01320-23)
Supplement: Fig. S8 — Identification of proteins interact directly with HtpA. [file mbio.01320-23-s0008.pdf]

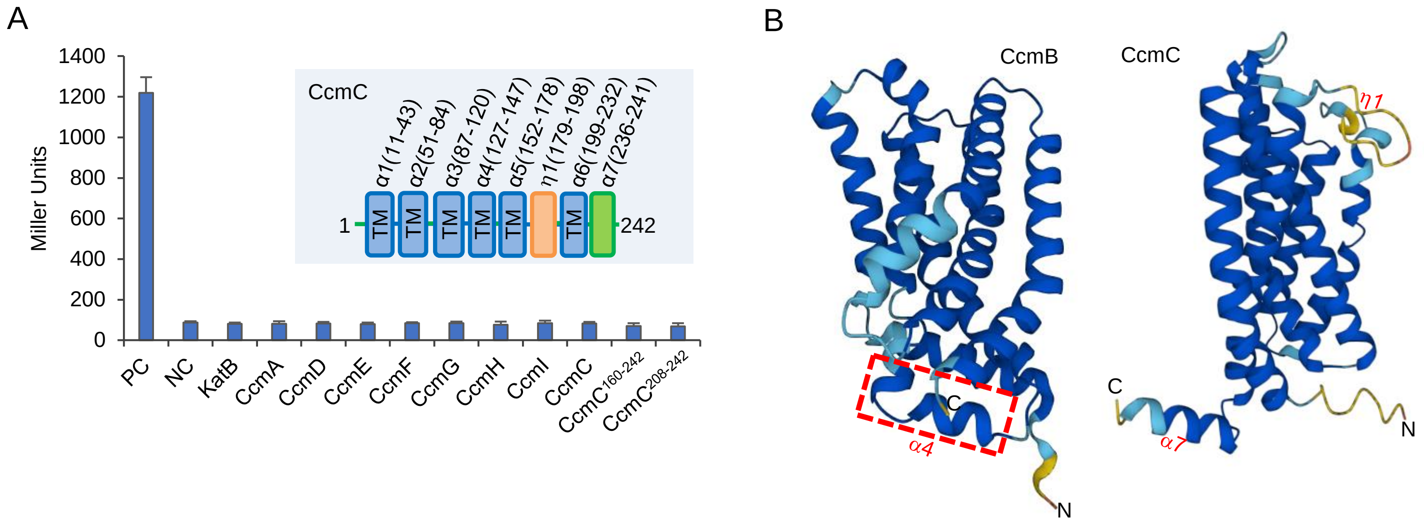

FIG S8. Identification of proteins that interact directly with HtpA. (A) Bacterial two-hybrid (BATCH) assay for detecting the interaction between HtpA and its targets. The interaction would activate expression of *lacZ* genes, which can be detected by β-galactosidase activity assay. The fast screening for interaction was performed with BTH101 reporter strains carrying various combination of vectors grown on LB plates containing 40 μg/ml X-gal and 0.5 mM IPTG at 30 ℃ for 24 hr. The activities of β-galactosidase then were quantified using cells in LB containing 40 μg/ml X-gal and 0.5 mM IPTG and presented as the average of four replicates and error bars in Miller Units. The secondary structure elements of CcmC was also shown, in which the elements in the cytoplasm and the periplasm are in green and orange respectively, and TM represents transmembrane helices. (B) The structure of CcmB and CcmC from AlphaFold Protein Structure Database. The critical helix is marked with a red box.
