# Supplementary material for: Bacterial TANGO2 homologs are heme-trafficking proteins that facilitate biosynthesis of cytochromes c
Source: mBio. 2023 Jul 18;14(4):e01320-23. doi: 10.1128/mbio.01320-23 (PMC10470608; doi:10.1128/mbio.01320-23)
Supplement: Table S1 — Proteins identified by BioID with HtpA as the bait in S. oneidensis. [file mbio.01320-23-s0009.pdf]

Table S1 Proteins identified by BioID with HtpA as the bait in *S. oneidensis*

Accession	Description	Coverage [%]	# Peptides	# PSMs	# Unique Peptides	# AAs	Abundances
QKG96549.1	methylcrotonoyl-CoA carboxylase	79	34	167	34	535	40381736030
Q8EBE6.1	Chaperone protein ClpB	70	48	109	48	857	15419307681
AAN54946.1	methylcrotonyl-CoA carboxylase biotin-binding subunit LiuD	68	36	55	36	694	3941027754
WP_011070682.1	ornithine decarboxylase SpeF	59	33	59	33	720	7152121224
WP_011071130.1	acetyl-CoA carboxylase	41	49	64	49	1517	2061151773
Q8EHT7.1	Chaperone protein DnaK	69	37	60	37	639	7179469042
Q8EFF7.1	Chaperone protein HtpG	58	32	71	32	637	11398900749
QKG96226.1	L-threonine dehydrogenase	79	21	51	21	382	14036995687
Q8EK70.1	Elongation factor Tu 2	71	20	70	1	394	109181954
QKG96372.1	acetyl-CoA C-acyltransferase	69	19	42	19	396	5098926188
Q8EK81.1	Elongation factor Tu 1	71	20	69	1	394	18125975014
QKG97100.1	NAD-glutamate dehydrogenase	33	46	52	46	1614	775964449.3
Q8EIJ7.1	Elongation factor G 2	49	28	44	27	697	2594375571
AAN56190.1	UDP-N-acetyl-d-glucosamine 6-dehydrogenase WbpA	70	26	46	26	426	3503026238
QKG96253.1	FAD-binding oxidoreductase	42	32	39	32	934	539664260
QKG96463.1	endopeptidase La	49	31	38	31	785	1564847805
Q8EHL5.1	Translation initiation factor IF-2	44	30	39	30	885	856868692.8
QKG95360.1	bifunctional aconitate hydratase 2/2-methylisocitrate dehydratase	49	33	39	33	865	916601823.3
QKG96937.1	30S ribosomal protein S1	51	26	37	26	555	1867913875
Q8E9Q5.1	Protein translocase subunit SecA	34	25	32	25	908	424854445
QKG97349.1	PrkA family serine protein kinase	53	30	38	29	644	1043982628
Q8EAG5.1	Adenylosuccinate synthetase	61	18	31	18	431	1142458022
QKG95000.1	DNA topoisomerase (ATP-hydrolyzing) subunit B	37	24	33	24	805	1179621953
QKG96949.1	ribonucleoside-diphosphate reductase subunit alpha	41	26	34	26	762	932805693.5
QKG97377.1	phosphate acetyltransferase	43	24	31	24	717	904636929.5
WP_011071022.1	chaperonin GroEL	59	25	32	25	545	2202975682
QKG95336.1	transcription termination factor Rho	54	22	32	22	421	1277618242
Q8E8C0.1	ATP synthase subunit beta	56	17	25	17	463	554275051

Q8E8B8.1	ATP synthase subunit alpha	61	28	31	28	513	1818772644
P82177.2	Malate dehydrogenase	70	18	28	18	311	2978767869
WP_011071723.1	GGDEF domain-containing protein	47	20	28	20	518	1660096796
QKG95098.1	NRDE family protein SO0126	65	11	82	11	266	7999476590
Q8ED70.1	DNA ligase	47	24	27	24	689	714317657.5
QKG96465.1	peptidylprolyl isomerase	45	24	30	24	621	332090639.5
QKG95352.1	pyruvate dehydrogenase (acetyl-transferring), homodimeric type	40	33	36	33	888	1015733221
QKG94720.1	response regulator transcription factor	81	14	23	14	225	1120020996
WP_011072933.1	SidA/IucD/PvdA family monooxygenase	40	14	22	14	498	391162827.9
QKG96893.1	type I glyceraldehyde-3-phosphate dehydrogenase	62	17	24	17	336	2063230638
WP_011071829.1	acyl-CoA dehydrogenase family protein	56	16	23	16	385	1251132998
Q8EFW4.1	Ribosomal RNA large subunit methyltransferase K/L	36	21	26	21	711	177117802.6
QKG98272.1	two-component system response regulator ArcA	69	14	23	14	238	2617030011
Q8E8A9.1	tRNA uridine 5-carboxymethylaminomethyl modification enzyme	37	19	23	19	629	481478971.5
QKG97274.1	ribonuclease E	22	17	22	17	1088	242292003.4
QKG97123.1	iron-sulfur cluster carrier protein ApbC	46	13	21	13	371	1192354476
QKG97017.1	acyl-CoA dehydrogenase	35	22	25	22	759	328605283
QKG96888.1	thiamine pyrophosphate-dependent dehydrogenase E1 component	53	20	28	20	392	1753081804
Q8EBQ9.1	CTP synthase	42	18	23	18	546	643222309
Q8E9U9.1	ATP-dependent protease ATPase subunit HslU	46	19	25	19	440	2709109938
QKG96268.1	isocitrate dehydrogenase	51	14	19	14	335	869069233
QKG94706.1	translational GTPase TypA	33	17	23	17	603	502198267.8
Q8EKR9.1	Fatty acid oxidation complex subunit alpha	34	20	22	20	716	183560024.8
QKG95354.1	dihydrolipoyl dehydrogenase	45	18	22	18	475	1141412469
WP_011071785.1	translation elongation factor Ts	58	14	22	14	283	1318707787
WP_011071416.1	redox-regulated ATPase YchF	49	13	21	13	363	631679697
QKG97482.1	IucA/IucC family siderophore biosynthesis protein	40	20	24	20	630	187875068.1
WP_011074420.1	tandem-95 repeat protein	24	21	23	4	1410	348284956
QKG98062.1	Re/Si-specific NAD(P)(+) transhydrogenase subunit alpha	41	14	18	14	508	248420255.5
QKG98149.1	M13 family peptidase	35	23	25	23	694	458033646
QKG94911.1	DNA polymerase I	26	20	22	20	922	181020389

WP_011073749.1	DEAD/DEAH box helicase	39	17	20	17	623	141254338.8
QKG97628.1	chemotaxis protein CheW	89	12	20	12	164	812751973.5
Q8EHT6.1	Chaperone protein DnaJ	43	14	19	14	378	755582201.5
Q8EH83.1	Elongation factor 4	33	16	20	16	596	303630397.1
WP_011074419.1	tandem-95 repeat protein	17	19	21	2	1408	2343866
QKG98360.1	Flp pilus assembly complex ATPase component TadA	40	18	20	18	586	240584275
QKG96846.1	acetolactate synthase 3 large subunit	31	15	17	15	572	381167336.5
QKG96489.1	DUF3450 domain-containing protein	58	12	16	12	260	979671811.3
Q8E8B9.1	ATP synthase gamma chain	61	13	17	13	286	351804516.3
WP_011073412.1	response regulator	53	12	16	12	237	572405336.3
WP_011073172.1	IMP dehydrogenase	46	18	21	18	488	279820317.3
Q8EK73.1	DNA-directed RNA polymerase subunit beta	16	23	24	23	1405	174617280.9
WP_011073286.1	recombinase RecA	39	11	15	11	357	230457158.5
QKG96889.1	alpha-ketoacid dehydrogenase subunit beta	51	10	14	10	325	606285168
QKG97531.1	MoxR family ATPase	54	12	16	12	318	161159821
Q8EGH5.1	30S ribosomal protein S2	38	8	15	8	242	789166826
QKG97015.1	MurR/RpiR family transcriptional regulator	52	10	14	10	284	211882250
QKG94881.1	two-component system response regulator OmpR	57	13	20	13	241	1477118210
WP_011072162.1	nickel-dependent hydrogenase large subunit	29	13	16	13	567	447983686
Q8EK47.1	DNA-directed RNA polymerase subunit alpha	62	18	22	18	329	552052680
QKG97267.1	3-oxoacyl-ACP reductase FabG	50	11	15	11	248	776473989.2
QKG96890.1	dihydrolipoyllysine-residue acetyltransferase	29	12	16	12	535	761140100.3
WP_011073034.1	FAD-binding protein	47	8	11	8	308	225062861.5
WP_011072028.1	succinate dehydrogenase flavoprotein subunit	28	16	21	16	588	426075510.4
AAN56210.2	flagellar polar localization control system FlhF inhibitory ATPase FlhG	56	12	16	12	293	775639022
QKG97579.1	electron transfer flavoprotein subunit beta/FixA family protein	52	10	13	10	249	288546437.3
QKG98057.1	hypothetical protein HRJ35_20020	29	14	18	14	578	166698722
QKG97617.1	dTDP-glucose 4,6-dehydratase	45	10	13	10	375	130964420.8
Q8EG20.1	Trigger factor	35	12	14	12	434	402289777
QKG97672.1	protein-glutamate O-methyltransferase CheR	60	13	16	13	279	185426501.3
Q8EK74.1	DNA-directed RNA polymerase subunit beta	16	23	23	23	1345	291974823.9

QKG95209.1	heme ABC transporter permease CcmC	34	21	18	26	248	2646435436
QKG97551.1	protein translocase subunit SecD	23	13	15	13	616	139981114.9
WP_011073167.1	cytochrome ubiquinol oxidase subunit I	27	13	17	13	518	2003886872
Q8EK54.1	50S ribosomal protein L6	66	8	12	8	177	205732653.5
P59320.1	Acetylornithine aminotransferase	37	9	13	9	405	95834168
WP_011073284.1	alanine--tRNA ligase	20	16	16	16	874	101692255.3
QKG96053.1	RNA polymerase sigma factor RpoD	24	14	16	14	619	490508399.9
Q8EC36.1	GTPase Der	37	14	15	14	487	317268142.5
WP_011071865.1	phosphate signaling complex protein PhoU	47	10	13	10	236	196324250
WP_011070853.1	SDR family NAD(P)-dependent oxidoreductase	48	7	10	7	225	204258820.1
WP_011074389.1	GNAT family N-acetyltransferase	45	6	12	6	175	508090919
WP_011071536.1	Hpt domain-containing protein	65	5	10	5	121	224110442.1
QKG94965.1	oxygen-independent coproporphyrinogen III oxidase	33	14	17	14	458	704140767.3
QKG97605.1	asparagine synthase (glutamine-hydrolyzing)	29	17	17	17	643	114435081
WP_011073972.1	hydroxymethylbilane synthase	49	11	15	11	310	102648390.8
Q8EK99.1	Selenide, water dikinase	38	8	10	8	352	57707370
WP_011073598.1	glutamyl-tRNA reductase	34	12	15	12	416	471695051
WP_011073713.1	response regulator	49	9	13	9	209	137496368.1
QKG95517.1	cAMP-activated global transcriptional regulator CRP	54	11	17	11	211	1550425493
WP_011072101.1	tetratricopeptide repeat protein	39	7	10	7	287	145871632
Q8EDI9.1	Uracil phosphoribosyltransferase	54	8	12	8	208	761965235.5
Q8EEZ1.1	Asparagine--tRNA ligase	32	15	17	15	466	167342629.5
QKG97616.1	glucose-1-phosphate thymidylyltransferase RfbA	40	12	17	12	304	299446630.3
Q8EJQ5.1	ATP-dependent RNA helicase RhlB	29	12	15	12	439	288249083.9
QKG98533.1	Na(+)-translocating NADH-quinone reductase subunit A	34	11	11	11	444	126718185
WP_011071159.1	pantoate--beta-alanine ligase	49	8	10	8	281	99854569
WP_011072267.1	transcription-repair coupling factor	12	13	16	13	1164	80339374.5
WP_011072385.1	YjjI family glycine radical enzyme	21	10	13	10	519	601861222
Q8EBB7.2	Formate-dependent phosphoribosylglycinamide formyltransferase	31	10	14	10	391	824334373
WP_011071949.1	tetratricopeptide repeat protein	28	9	12	9	415	328588801.5
Q8EK19.1	3-dehydroquinate synthase	39	11	12	11	359	113064065

WP_011071100.1	CBS domain-containing protein	81	8	11	8	141	525419567
QKG98007.1	heme utilization cystosolic carrier protein HutX	55	8	13	8	186	856081424.5
QKG95357.1	M13 family peptidase	19	11	14	11	680	937916678.5
WP_011072149.1	phenylalanine--tRNA ligase subunit alpha	47	16	19	16	327	721461326
Q8EGP3.1	Recombination-associated protein RdgC	36	11	14	11	304	191970837.3
QKG96901.1	universal stress protein UspE	28	8	12	8	310	689352793
Q8EKN9.1	Glycerol-3-phosphate dehydrogenase [NAD(P)+]	37	8	11	8	338	64747712.25
P59183.1	30S ribosomal protein S3	64	14	15	14	230	1241406718
QKG98203.1	DUF2333 family protein	46	12	14	12	322	178936644
QKG97635.1	RNA polymerase sigma factor FliA	37	6	9	6	237	14690432.5
P59124.1	30S ribosomal protein S5	60	10	14	10	167	424302910.5
QKG94672.1	DUF3014 domain-containing protein	38	7	9	7	297	13459300.5
WP_011071477.1	HDOD domain-containing protein	43	11	13	11	279	207628750.5
Q8EF99.1	Phenylalanine--tRNA ligase beta subunit	20	13	13	13	795	123201065.3
5K8C	A Chain A, 3-deoxy-alpha-D-manno-octulosonate 8-oxidase	35	10	12	10	358	224150225.5
QKG97314.1	DEAD/DEAH box helicase	27	11	12	11	449	205998981.5
WP_172966560.1	ketoacyl-ACP synthase III	35	10	11	10	319	377402287
QKG96656.1	hypothetical protein HRJ35_11970	44	6	9	6	184	243459850.5
Q8EAZ2.1	Bifunctional protein HldE	29	12	13	12	476	341406847
WP_011073976.1	retention module-containing protein	7	10	12	10	2768	73462021.25
QKG98262.1	serine protein kinase RIO	36	6	8	6	285	51779223.75
QKG97797.1	ribosome-associated translation inhibitor RaiA	79	7	11	5	118	556026494.5
WP_011072785.1	M48 family metalloprotease	30	13	14	13	489	119264618.5
WP_011072087.1	bifunctional UDP-sugar hydrolase/5'-nucleotidase UshA	22	12	13	12	569	238007867.3
Q8ECI8.1	Proline--tRNA ligase	26	12	13	12	570	83198749.61
QKG96376.1	enoyl-CoA hydratase/isomerase family protein	38	14	15	4	383	296474880
QKG97561.1	CIA30 family protein	41	5	11	5	174	158409967.5
WP_011071395.1	serine hydrolase	28	8	10	8	391	113695214.8
QKG95213.1	heme lyase CcmF/NrfE family subunit	24	7	9	8	659	14345544
Q8EI18.1	dimethylbenzimidazole phosphoribosyltransferase	19	6	9	6	350	183369132
WP_011074184.1	signal recognition particle-docking protein FtsY	24	11	12	11	510	2406329034

QKG95659.1	OsmC domain/YcaO domain-containing protein	20	13	13	13	732	25518667
QKG95129.1	type II secretion system ATPase GspE	27	14	15	13	521	113722404.3
7L4S	Transcriptional regulator of oxidative stress OxyR	40	9	11	9	304	181814533.8
WP_011073807.1	type II secretion system GspH family protein	52	5	8	5	171	507003329.3
P59188.1	Protein FdhE homolog	39	8	10	8	302	52673630.13
QKG96550.1	isovaleryl-CoA dehydrogenase	31	8	8	8	389	137593644
QKG97517.1	beta-ketoacyl-ACP synthase I	34	10	10	10	411	100130587.5
QKG95128.1	type II secretion system secretin GspD	20	12	13	12	704	59548463.25
QKG96579.1	dihydrolipoyllysine-residue succinyltransferase	29	9	11	9	395	205248040.5
QKG95881.1	FKBP-type peptidyl-prolyl cis-trans isomerase	45	9	10	9	255	275301320
QKG94959.1	molybdopterin-guanine dinucleotide biosynthesis adaptor protein	18	8	9	8	599	37433593.25
Q8EJ54.1	N-succinylglutamate 5-semialdehyde dehydrogenase	25	9	9	9	487	68148263.5
AAN56753.2	SH3 domain protein	52	6	7	6	192	37101645
QKG97389.1	DUF3083 family protein	29	8	9	8	355	112168071.8
Q8EGR9.1	1-deoxy-D-xylulose-5-phosphate synthase	21	10	11	10	622	47894663.75
Q8EER3.1	DNA translocase FtsK	14	11	12	11	911	62028900.5
QKG98018.1	universal stress protein	73	6	7	6	143	227202471
WP_011071411.1	PhoH family protein	34	11	11	10	362	160993207.7
Q8EIK8.1	Glutathione synthetase	44	9	10	9	315	39286798.5
WP_011070608.1	50S ribosomal protein L1	40	8	11	8	233	366424884.9
Q8EAF7.1	UDP-N-acetylglucosamine 1-carboxyvinyltransferase	20	7	9	7	419	158259934
QKG96239.1	hypothetical protein HRJ35_09590	24	12	13	12	503	134826017.8
QKG94740.1	electron transfer flavoprotein-ubiquinone oxidoreductase	26	13	13	13	549	111727523.5
QKG96452.1	Fe(2+) transporter permease subunit FeoB	17	12	13	11	764	210097793.8
QKG97848.1	thiamine-phosphate kinase	37	8	11	8	318	418528142.6
WP_011070812.1	DUF853 family protein	23	8	8	8	493	44350570.25
QKG97374.1	formate C-acetyltransferase	12	8	10	8	760	32084517.75
WP_011070606.1	transcription termination/antitermination protein	50	7	9	7	183	951095322.3
QKG94879.1	RNA-binding transcriptional accessory protein	13	10	11	10	784	47556411.5
QKG98019.1	STAS/SEC14 domain-containing protein	50	6	9	6	132	2797004144
QKG95353.1	dihydrolipoyllysine-residue acetyltransferase	15	8	8	8	677	127493539

WP_011073187.1	histidine--tRNA ligase	23	8	9	8	425	23836314
QKG98488.1	c-type cytochrome biogenesis protein CcmI	27	10	10	10	415	67591845
Q8EHL4.1	Ribosome-binding factor A	59	7	10	7	147	249379075.8
WP_011071433.1	transcription termination factor NusA	22	11	12	11	499	138177625.6
QKG98115.1	hypoxanthine phosphoribosyltransferase	47	7	8	7	176	178275715
3JU1	B Chain B, Enoyl-CoA hydratase/isomerase family protein	27	11	12	1	407	34722316
QKG96258.1	(2E,6E)-farnesyl diphosphate synthase	21	4	6	4	293	16106014
Q8EBR6.1	Protein-L-isoaspartate O-methyltransferase	55	9	9	9	211	99338901.5
QKG96034.1	sulfurtransferase	31	7	8	7	285	74937327.75
WP_011073899.1	UDP-N-acetylmuramate--L-alanine ligase	12	5	9	5	488	69131715
Q8EB83.1	GTPase Obg	29	9	9	9	388	200626618.8
AAN52943.2	type II restriction-modification system N4-cytosine or N6-adenine DNA methyltransferase (plasmid)	22	11	11	11	568	150776487
QKG95274.1	branched-chain amino acid aminotransferase	23	7	8	7	363	32460797.75
Q8EAR3.1	Peptide chain release factor 1	25	8	10	8	363	57367472
Q8EK40.1	Cytochrome c biogenesis ATP-binding export protein CcmA	9	1	1	1	216	63689656
Q8EKT6.1	Membrane protein insertase YidC	20	7	7	7	541	36620032
QKG97933.1	bifunctional 2',3'-cyclic-nucleotide 2'-phosphodiesterase/3'-nucleotidase	17	11	11	10	685	27163655.75
WP_011071557.1	ribonuclease III	38	8	9	8	226	146013470
Q8EK59.1	50S ribosomal protein L14	68	8	10	8	122	396483304
WP_011071592.1	DEAD/DEAH box helicase	27	11	12	10	433	97077066
Q8EHU2.1	Glutamate 5-kinase	25	9	10	9	372	199912864
WP_011073974.1	uroporphyrinogen-III C-methyltransferase	20	7	8	7	414	70883498
WP_011071565.1	signal recognition particle protein	16	6	8	6	457	33806694.56
Q8EDI8.2	Phosphoribosylformylglycinamidine cyclo-ligase	22	5	7	5	345	97443080.38
QKG98376.1	paraslipin	27	8	9	8	311	175023377
Q8EHQ6.1	Lipoyl synthase	23	8	9	8	321	67520919.38
Q8EE14.1	Probable septum site-determining protein MinC	42	7	8	7	221	66169076.5
QKG97565.1	DEAD/DEAH box helicase	23	9	9	9	427	75713670
WP_011071632.1	dimethylsulfoxide reductase subunit B	43	7	8	7	224	28939982.63
QKG98523.1	ATP-dependent RNA helicase SrmB	30	12	12	11	420	112263669.3
Q8EEE7.1	Carboxy-S-adenosyl-L-methionine synthase	23	4	6	4	243	41484515.25

WP_011071394.1	DUF493 family protein YbeD	86	7	8	7	88	216404238
QKG94635.1	heme biosynthesis protein HemY	18	7	9	6	388	39267052
QKG96139.1	hypothetical protein HRJ35_09015	27	7	9	7	308	88300298
QKG98063.1	Re/Si-specific NAD(P)(+) transhydrogenase subunit beta	24	7	8	7	469	24864147
QKG95646.1	FAD-dependent 2-octaprenylphenol hydroxylase	21	8	9	8	407	86712914.38
WP_011073010.1	S-adenosylmethionine ribosyltransferase-isomerase QueA	27	7	8	7	345	84336452.88
AAN54063.1	transcriptional regulator LysR family	22	4	5	4	296	10594059
WP_011070634.1	heme exporter protein CcmD	21	4	7	6	66	18141396
QKG98205.1	enoyl-CoA hydratase	21	3	6	3	245	32110719.19
QKG98090.1	TIGR00153 family protein	31	7	10	7	226	696235472.5
WP_011073378.1	hemerythrin domain-containing protein	42	7	10	7	178	101766959.5
Q8ED69.1	Cell division protein ZipA	18	5	8	5	344	80939577
WP_011071526.1	tyrosine--tRNA ligase	20	7	8	7	398	101277401.5
QKG97788.1	DEAD/DEAH box helicase	20	7	8	7	409	296266099.6
QKG95501.1	ubiquinol-cytochrome c reductase iron-sulfur subunit	34	6	8	6	196	192341863.3
QKG97151.1	response regulator transcription factor	26	4	6	4	220	34527491.5
QKG98196.1	DNA topoisomerase IV subunit B	18	9	9	9	628	60552921
WP_011072043.1	response regulator	36	7	8	7	224	28232264.25
WP_011074449.1	S-(hydroxymethyl)glutathione dehydrogenase	29	9	9	9	376	183447068
QKG97633.1	protein phosphatase CheZ	30	5	6	5	245	42340630.25
QKG95214.1	DsbE family thiol:disulfide interchange protein	58	8	8	8	184	75606532
WP_011073982.1	EAL domain-containing protein	15	8	8	8	639	88590662
QKG98004.1	long-chain fatty acid--CoA ligase	14	6	7	6	532	9608304
WP_011072519.1	AsmA family protein	13	7	8	7	606	66181144.5
QKG97257.1	nucleotide-binding protein	23	5	7	5	271	74833619
WP_011072540.1	ribosome biogenesis GTPase YlqF	27	8	9	8	313	43907746.25
QKG97474.1	phosphoribosylanthranilate isomerase TrpF	21	8	8	8	498	43057607
QKG95713.1	PAS domain S-box protein	6	8	9	8	1765	16066606.3
QKG96006.1	PilZ domain-containing protein	10	6	8	6	792	8718191.625
WP_011071697.1	LUD domain-containing protein	29	4	5	4	189	51796123.5
QKG98367.1	MSHA biogenesis protein MshI	28	7	8	7	292	43017938.5

QKG96577.1	succinate dehydrogenase iron-sulfur subunit	36	10	11	10	235	140717011.5
WP_011071409.1	CNNM family magnesium/cobalt transport protein CorC	30	8	8	8	291	86856746
WP_011073298.1	nucleoside triphosphate pyrophosphohydrolase	21	4	5	4	312	7741633.75
Q8EK67.1	50S ribosomal protein L4	35	4	5	4	201	4386724.688
QKG98198.1	3',5'-cyclic-AMP phosphodiesterase	28	5	6	5	278	69394529
QKG96474.1	phage shock protein PspA	34	6	6	6	227	34789589.5
WP_011074159.1	diguanylate cyclase PdgA	15	10	10	10	705	70976658.5
QKG96341.1	outer membrane protein assembly factor BamA	10	7	7	7	826	16511354
WP_011072514.1	LysR substrate-binding domain-containing protein	22	5	6	5	299	12236902.5
WP_011072348.1	cytochrome-c oxidase, cbb3-type subunit III	24	6	8	6	322	156470685.5
Q8EEH8.1	3-phosphoshikimate 1-carboxyvinyltransferase	20	6	7	6	426	112578913
QKG96864.1	leucine-responsive transcriptional regulator Lrp	45	7	8	7	168	558636154
QKG97719.1	tetratricopeptide repeat protein	35	6	7	6	206	36884313.5
QKG97932.1	M3 family metallopeptidase	12	6	6	6	716	10970881.13
Q8E9R7.1	Ubiquinone/menaquinone biosynthesis C-methyltransferase UbiE	31	7	8	7	251	326128050
Q8E8Q9.1	Glycerol-3-phosphate acyltransferase	10	9	10	9	809	38625970.25
QKG97134.1	NADP-dependent isocitrate dehydrogenase	11	7	7	7	741	99349134
QKG96778.1	CreA family protein	39	4	5	4	165	84568339
QKG95490.1	hypothetical protein HRJ35_05420	49	8	9	8	151	445606175
QKG95515.1	response regulator transcription factor	33	6	7	6	224	63493171.5
WP_011073076.1	NAD-dependent epimerase/dehydratase family protein	19	3	4	3	340	19739644.25
AAN56968.2	uncharacterized protein SO_3994	23	7	7	7	386	63210623
AAN52942.1	type II restriction-modication system restriction endonuclease	26	8	8	8	348	55611589.5
Q8ECD7.1	Protein-glutamate methylesterase/protein-glutamine glutaminase 1	15	4	5	4	374	600234165.3
QKG96945.1	DNA gyrase subunit A	9	9	10	9	919	147744189
QKG95500.1	protease modulator HflC	35	11	11	11	297	160606932
QKG96375.1	enoyl-CoA hydratase	29	9	9	9	257	273565794
QKG94791.1	ligand-gated channel protein	12	5	6	5	663	4572842.5
Q8EEG9.1	Ubiquinone biosynthesis O-methyltransferase	31	5	5	5	236	16345739.25
Q8EF25.1	Nucleoid-associated protein SO_2177	22	4	5	4	342	7669834
QKG96726.1	acetate--CoA ligase family protein	9	6	6	6	903	47793363.38

QKG97888.1	NAD(P)/FAD-dependent oxidoreductase	14	5	6	5	429	132888362.3
WP_011074105.1	response regulator	35	7	7	7	228	209474473
Q8E8J7.1	Probable GTP-binding protein EngB	25	5	8	5	219	34575819
Q8EK65.1	50S ribosomal protein L2	24	7	9	7	274	83784711
QKG97521.1	ATP-NAD kinase family protein	20	6	6	6	376	20909097.75
WP_011074032.1	tryptophan 7-halogenase	16	5	5	5	436	36827391
QKG97903.1	bifunctional riboflavin kinase/FAD synthetase	24	6	6	6	311	31813655
QKG95263.1	hypothetical protein HRJ35_04160	20	4	5	4	307	12902587.5
Q8EIB4.1	S-adenosylmethionine synthase	16	5	6	5	383	60385005
QKG96251.1	lactate utilization protein	14	6	7	6	464	56803776.25
Q8EAG8.1	23S rRNA (guanosine-2'-O-)-methyltransferase RlmB	25	5	7	5	246	26452148.5
QKG94909.1	cytochrome c4	23	6	7	6	207	81104922
Q8EK51.1	50S ribosomal protein L15	54	7	7	7	144	148295708
QKG96252.1	(Fe-S)-binding protein	35	7	7	7	247	104809853
Q8EKP0.1	Protein-export protein SecB	44	5	6	5	157	511315968
QKG96185.1	cytoplasmic protein	18	6	6	6	358	36449412
QKG96845.1	acetolactate synthase small subunit	37	5	5	5	164	69305047.75
WP_011073988.1	accessory factor UbiK family protein	75	6	6	6	84	326910743
Q8EKD3.1	Phosphoenolpyruvate carboxykinase (ATP)	14	5	5	5	513	14930341
Q8EKS0.1	3-ketoacyl-CoA thiolase	12	4	5	4	387	72561598
WP_011071738.1	NAD(P)-binding protein	6	5	7	5	1041	13494991.75
QKG98406.1	DUF971 domain-containing protein	44	4	5	4	128	120592309
QKG96807.1	ATP-dependent RNA helicase HrpA	6	9	10	9	1293	34616237.5
QKG97928.1	insulinase family protein	8	7	7	7	949	40958775.75
WP_011071650.1	N-6 DNA methylase	11	7	7	7	684	29243941.5
QKG97325.1	GntR family transcriptional regulator	37	6	6	6	223	60422447.5
WP_011073726.1	response regulator	20	6	6	6	357	29877187.75
P59375.1	30S ribosomal protein S11	59	6	6	6	130	118123798
QKG95911.1	NADH:ubiquinone reductase (Na(+)-transporting) subunit F	17	7	7	7	418	89130026.5
QKG95228.1	type IV pilus secretin PilQ family protein	10	6	7	6	684	20410006
WP_011073577.1	UDP-N-acetylmuramate ligase	12	4	5	4	465	36831245.5

Q8EGS0.1	Protein GrpE	34	7	7	7	206	156039679
Q8EI31.1	NADH-quinone oxidoreductase subunit C/D	11	6	6	6	601	19640987.5
WP_011074409.1	ParA family protein	16	6	6	6	399	702483432.5
QKG96513.1	ABC transporter ATP-binding protein	10	7	7	7	640	116387077
P59131.1	30S ribosomal protein S4	30	7	8	7	206	270161689.5
WP_011073869.1	c-di-GMP binding protein MxdA	14	5	5	5	462	18558348.5
WP_011070429.1	HDOD domain-containing protein	19	5	6	5	347	62911646.31
QKG95213.1	heme lyase CcmF/NrfE family subunit	9	4	4	4	659	3545342
QKG95499.1	FtsH protease activity modulator HflK	16	6	6	6	381	118433856
WP_011072456.1	pyruvate kinase	17	6	6	6	479	57362335
WP_011072669.1	LuxR C-terminal-related transcriptional regulator	31	6	6	6	211	121490744
QKG97856.1	energy-dependent translational throttle protein EttA	14	7	7	7	555	54266889.5
QKG96547.1	hydroxymethylglutaryl-CoA lyase	17	5	6	5	315	41478261.5
WP_011073078.1	Wzz/FepE/Etk N-terminal domain-containing protein	21	6	6	6	329	41960988.5
AAN54132.2	TIGR02722 family lipoprotein	28	4	4	4	201	44540289
QKG96293.1	RsmB/NOP family class I SAM-dependent RNA methyltransferase	15	7	7	7	458	97098772.5
WP_011073218.1	ferrochelatase	20	5	5	5	327	8589957
