# Supplementary material for: Bacterial TANGO2 homologs are heme-trafficking proteins that facilitate biosynthesis of cytochromes c
Source: mBio. 2023 Jul 18;14(4):e01320-23. doi: 10.1128/mbio.01320-23 (PMC10470608; doi:10.1128/mbio.01320-23)
Supplement: Table S2 — Strains, plasmids, primers used in this study. [file mbio.01320-23-s0010.pdf]

Table S2A. Strains and plasmids used in this study

Strain or plasmid	description	Source/reference
Strains		
E.coli		
DH5α	Host strain for plasmids	Lab stock
WM3064	Donor strain for conjugation; Δ*dapA*	W. Metcalf, UIUC
BTH101	Reporter strain for Bacterial Adenylate Cyclase Two-Hybrid (BACTH)	EUROMEDEX
ER2738	Host strain for intermediate construction in BACTH	Lab stock
S. oneidensis		
MR-1	Wild type	Lab stock
HG0265	Δ*ccmI* derived from MR-1	(23)
HG0266	Δ*ccmF* derived from MR-1	(29)
HG1937	Δ*fur* derived from MR-1	(70)
HG1070	Δ*katB* derived from MR-1	(43)
HG3286-5	Δ*cydAB* derived from MR-1	(46)
HG0126	Δ*htpA* derived from MR-1	This study
HG0126-0265	Δ*ccmI*Δ*htpA* derived from MR-1	This study
Plasmids		
pHGM01	Ap^r Gm^r Cm^r suicide vector	(29)
pHGT01	Gm^r, mariner-based transposon vector	(30)
pHGI01	Km^r, integrative *lacZ* reporter system	(46)
pHGEN-P*tac*	IPTG-inducible P*tac* expression vector	(59)
pHGEN-P*tac*-HtpA	inducible expression of HtpA	This study
pHGEN-P*tac*-HtpA$^{strep\ tag\text{-}II}$	inducible expression of HtpA$^{strep\ tag\text{-}II}$	This study
pHGEN-P*tac*-HtpA (Y108A)$^{strep\ tag\text{-}II}$	inducible expression of HtpA(Y108A)$^{strep\ tag\text{-}II}$	This study
pHGEN-P*tac*-HtpA^{H11A}	inducible expression of HtpA^{H11A}	This study
pHGEN-P*tac*-HtpA^{Y14A}	inducible expression of HtpA^{Y14A}	This study
pHGEN-P*tac*-HtpA^{H26A}	inducible expression of HtpA^{H26A}	This study
pHGEN-P*tac*-HtpA^{H27A}	inducible expression of HtpA^{H27A}	This study
pHGEN-P*tac*-HtpA^{C97A}	inducible expression of HtpA^{C97A}	This study
pHGEN-P*tac*-HtpA^{H104A}	inducible expression of HtpA^{H104A}	This study
pHGEN-P*tac*-HtpA^{Y108A}	inducible expression of HtpA^{Y108A}	This study
pHGEN-P*tac*- HtpA^{Y108F}	inducible expression of HtpA^{Y108F}	This study
pHGEN-P*tac*- HtpA^{H139A}	inducible expression of HtpA^{H139A}	This study
pHGEN-P*tac*- HtpA^{Y207A}	inducible expression of HtpA^{Y207A}	This study
pHGEN-P*tac*- HtpA^{H210A}	inducible expression of HtpA^{H210A}	This study
pHGEN-P*tac*- HtpA^{Y213A}	inducible expression of HtpA^{Y213A}	This study
pHGEN-P*tac*- HtpA^{Y236A}	inducible expression of HtpA^{Y236A}	This study
pHGEN-P*tac*- HtpA^{H249A}	inducible expression of HtpA^{H249A}	This study
pHGEN-P*tac*- HtpA^{C262A}	inducible expression of HtpA^{C262A}	This study
pHGEN-P*tac*-*Tc*HtpA$^{strep\text{-}tag\text{-}II}$	inducible expression of *T. crassostreae* homologue (WP068544776.1)	This study
pHGEN-P*tac*-*Pa*HtpA$^{strep\text{-}tag\text{-}II}$	inducible expression of *P. aeruginosa* homologue (WP058199997.1)	This study
pHGEN-P*tac*-*Dm*TANGO2$^{strep\text{-}tag\text{-}II}$	inducible expression of *D. melanogaster* TANGO2 (NP001259525.1)	This study
pHGEN-P*tac*-*Sc*TANGO2$^{strep\text{-}tag\text{-}II}$	inducible expression of *S. cerevisiae* TANGO2 (NP011643.1)	This study
pHGEN-P*tac*-*At*TANGO2$^{strep\text{-}tag\text{-}II}$	inducible expression of *A. thaliana* TANGO2 (NP568038.1)	This study
pHGEN-P*tac*-*Hs*TANGO2$^{strep\text{-}tag\text{-}II}$	inducible expression of *H. sapiens* TANGO2 (NP001270035.1)	This study
pHGEN-P*tac*-*HtpA*-*BirA**	biotinylating the proteins interacting with HtpA	This study
pKT25	Km^r, IPTG-inducible co-expression with T25 fragment vector	EUROMEDEX
pUT18C	Am^r, IPTG-inducible co-expression with T18 fragment vector	EUROMEDEX
pKT25-zip	GCN4 and T25 fragment fusion proteins expression, positive control for BACTH	EUROMEDEX
pUT18C-zip	GCN4 and T18 fragment fusion proteins expression, positive control for BACTH	EUROMEDEX
pKT25-*KatB*	KatB and T25 fusion proteins expression	This study
pKT25-*HtpA*	HtpA and T25 fusion proteins expression	This study
pUT18C-*KatB*	KatB and T18 fusion proteins expression	This study
pUT18C-*HtpA*	HtpA and T18 fusion proteins expression	This study
pKT25-*CcmA*	CcmA and T25 fusion proteins expression	This study
pKT25-*CcmB*	CcmB and T25 fusion proteins expression	This study
pKT25-*CcmC*	CcmC and T25 fusion proteins expression	This study
pKT25-*CcmD*	CcmD and T25 fusion proteins expression	This study
pKT25-*CcmE*	CcmE and T25 fusion proteins expression	This study
pKT25-*CcmF*	CcmF and T25 fusion proteins expression	This study
pKT25-*CcmG*	CcmG and T25 fusion proteins expression	This study
pKT25-*CcmH*	CcmH and T25 fusion proteins expression	This study
pKT25-*CcmI*	CcmI and T25 fusion proteins expression	This study
pKT25-*CcmB*v	CcmB truncations and T25 fusion proteins expression	This study
pKT25-*CcmC*v	CcmC truncations and T25 fusion proteins expression	This study
pHGEN-P*tac*-HtpA-*GFP*	inducible expression of HtpA and GFP fusion proteins expression	This study
pHGEN-P*tac*-*FlhG*-*GFP*	inducible expression of FlhG and GFP fusion proteins expression	This study
pHGEN-P*tac*-*PetA*-*GFP*	inducible expression of PetA and GFP fusion proteins expression	This study
pHGI01-PHtpA	promoter activity assays of HtpA based on the Integrative *lacZ* reporter system	This study
pHGI01-PSO0127	promoter activity assays of SO0127 based on the Integrative *lacZ* reporter system	This study
pHGI01-P*hemA*	promoter activity assays of *HemA* based on the Integrative *lacZ* reporter system	This study
pHGI01-P*hemC*	promoter activity assays of *HemC* based on the Integrative *lacZ* reporter system	This study
pHGI01-P*hemG*	promoter activity assays of *HemG* based on the Integrative *lacZ* reporter system	This study
pHGI01-P*hemH*	promoter activity assays of *HemH* based on the Integrative *lacZ* reporter system	This study

Ap^r, apramycin resistance; Gm^r, gentamicin resistance; Cm^r, chloramphenicol resistance; Km^r, kanamycin resistance.

Table S2B. Primers used in this study

Primers	Sequence
In-frame deletion	
HtpA-5O	GGGGACAAGTTTGTACAAAAAAGCAGGCTTGCTCAGCTTAGTGCTAGTG
HtpA-SI	GCGCCGTGAATAAATCTACACCCGATTCGCACAAATAATCA
HtpA-3O	GGGGACCACTTTGTACAAGAAAGCTGGGTCAGCGGCATAAACGTGTAAA
HtpA-3I	TGTAGATTTATTCACGGCGCGAGCCAGACTGTTTAGTGGT
HtpA-LF	GATTACCGATCCCCACACAA
HtpA-LR	GAGCGAGGACACCAATGATA
HtpA-SF	TCATTGGGTGAGCTGAAGTT
HtpA-SR	TCATTGGGTGAGCTGAAGTT
Inserted segment verification	
pHGEN-P*tac*-F	ACCGGAGCTGTTGACAATTA
pHGEN-P*tac*-R	CGGCGTTTCACTTCTG
pUT18-F	GCGAGGGCTATGTCTTCTACG
pUT18-R	GGGCTGGCTTAACTATGCGG
pKT25-F	CGCATCTGTCCAACTTCCGC
pKT25-R	CGCCAGGGTTTTCCCAGTCA
pHGI01-F	AGCAGGATTCCCGTTGAG
pHGI01-R	GTAACAACCCGTCGGATTCT
Controlled expression	
pHGEN-P*tac*-HtpA-F	CCGGAATTCATGTGCATACTCTTCATCGCG
pHGEN-P*tac*-HtpA-R	CGCGGATCCCTAATGATGATGATGATGATGAAAAACCACTAAACAGTCTGGCT
pHGEN-P*tac*-HtpA$^{\text{Strep-tag-II}}$-F	CCG GAATTC ATGTGCATACTCTTCATCGC
pHGEN-P*tac*-HtpA$^{\text{Strep-tag-II}}$-R	CGC GGATCC CTA TTTTTCGAACTGAGGGTGAGACCA AAAAACCACTAAACAGTCTGGC
pHGEN-P*tac*-HtpA(Y108A$^{\text{)Strep-tag-II}}$-F	CCG GAATTC ATGTGCATACTCTTCATCGC
pHGEN-P*tac*-HtpA(Y108A)$^{\text{Strep-tag-II}}$-R	CGC GGATCC CTA TTTTTCGAACTGAGGGTGAGACCA AAAAACCACTAAACAGTCTGGC
pHGEN-P*tac*-HtpA-*GFP*-F	CCGGAATTCATGTGCATACTCTTCATCGCG
pHGEN-P*tac*-HtpA-*GFP*-R	CCCAAGCTT TCAAAGATCTACCATGTACAGCTCGT
pHGEN-P*tac*-*CcmI*-F	CCG GAATTC ATGACGACATTTTGGATTTTTATTGC
pHGEN-P*tac*-*CcmI*-R	CCC AAGCTT TTATTGTACTTGAGTATCCAG
Site-directed mutagenesis	
pHGEN-P*tac*-HtpA(H11A)-F	CTTCATCGCGCTTAATGCTGCTCCCCAGTATCCCTTGATTA
pHGEN-P*tac*-HtpA(H11A)-R	AGTATGCACATGAATTCTCCT
pHGEN-P*tac*-HtpA(Y14A)-F	GCTTAATGCTCATCCCCAGGCTCCCTTGATTATTTGTGCGA
pHGEN-P*tac*-HtpA(Y14A)-R	GCGATGAAGAGTATGCACAT
pHGEN-P*tac*-HtpA(H26A)-F	TGCGAATCGGGATGAGTTTGCCCACAGACCCACGGCACCAG
pHGEN-P*tac*-HtpA(H26A)-R	CAAATAATCAAGGGATACTG
pHGEN-P*tac*-HtpA(H27A)-F	GAATCGGGATGAGTTTCACGCCAGACCCACGGCACCAGCGC
pHGEN-P*tac*-HtpA(H27A)-R	GCACAAATAATCAAGGGATA
pHGEN-P*tac*-HtpA(C97A)-F	AAATTCTGGCTCGCTTATCGCCCCTAACTGGCTTGTGGAGC
pHGEN-P*tac*-HtpA(C97A)-R	AATGCCTGAATGACGAGTTC
pHGEN-P*tac*-HtpA(H104A)-F	CCCTAACTGGCTTGTGGAGGCCGCACAAGATTATCAACCCT
pHGEN-P*tac*-HtpA(H104A)-R	CAGATAAGCGAGCCAGAATT
pHGEN-P*tac*-HtpA(Y108A)-F	TTGTGGAGCACGCACAAGATGCTCAACCCTTCAACTTAGTCT
pHGEN-P*tac*-HtpA(Y108A)-R	GCCAGTTAGGGCAGATAAGC
pHGEN-P*tac*-HtpA(Y108F)-F	TTGTGGAGCACGCACAAGATTTTCAACCCTTCAACTTAGTCT
pHGEN-P*tac*-HtpA(Y108F)-R	GCCAGTTAGGGCAGATAAGC
pHGEN-P*tac*-HtpA(H139A)-F	AAAGTTAACCCAAGGATTTGCTGCTATTAGCAATGGGGCAA
pHGEN-P*tac*-HtpA(H139A)-R	ACCGTCTCCCGCCTAATACT
pHGEN-P*tac*-HtpA(Y207A)-F	GCGCCGTTTAGCCGCGATTGCTATTCGTCACCCCGATTATG
pHGEN-P*tac*-HtpA(Y207A)-R	TTCCATTCTAGCCCCACACC
pHGEN-P*tac*-HtpA(H210A)-F	AGCCGCGATTTATATTCGTGCCCCCGATTATGGCACCCGAT
pHGEN-P*tac*-HtpA(H210A)-R	AAACGGCGCTTCCATTCTAG
pHGEN-P*tac*-HtpA(Y213A)-F	TTATATTCGTCACCCCGATGCTGGCACCCGATCCACCAGCA
pHGEN-P*tac*-HtpA(Y213A)-R	ATCGCGGCTAAACGGCGCTT
pHGEN-P*tac*-HtpA(Y236A)-F	TCAGTTCACCGAGGTGAGAGCCGATGGTAAGGGTCGGCGTC
pHGEN-P*tac*-HtpA(Y236A)-R	ATTTCCCCTTGGGCATTTTG
pHGEN-P*tac*-HtpA(H249A)-F	TCTGGGACAACAGGATTTCGCCTTCACCCTACCGCCCCAAT
pHGEN-P*tac*-HtpA(H249A)-R	CGCCGACCCTTACCATCGTA
pHGEN-P*tac*-HtpA(C262A)-F	ATTACCGCCAGAGCCAGACGCTTTAGTGGTTTTTTAGCTTA
pHGEN-P*tac*-HtpA(C262A)-R	TGGGGCGGTAGGGTGAAGTG
BATCH	
pUT18c-linerself-F	AATTCATCGATATAACTAAGTAAT
pUT18c-linerself-R	CGGTACCCGGGGATCCTCTAGAGT
pKU18C-KatB-F	ACTCTAGAGGATCCCCGGGTACCGATGAGTCAACAGTATTTAACCAGCC
pKU18C-KatB-R	ATTACTTAGTTATATATCGATGAATTTTATAACCCCAGCGCCATTTT
pKT25-KatB-F	CGG GGATCC ATGAGTCAACAGTATTTAACCAGCC
pKT25-KatB-R	CCG GAATTC TTATAACCCCAGCGCCATTTT
pTU18C-HtpA-F	ACTCTAGAGGATCCCCGGGTACCGATGTGCATACTCTTCATCGCG
pTU18C-HtpA-R	ATTACTTAGTTATATATCGATGAATTCTAAAAAACCACTAAACAGTCTGGC
pKT25-HtpA-F	CGG GGTACC ATGTGCATACTCTTCATCGCG
pKT25-HtpA-R	CCG GAATTC CTAAAAAACCACTAAACAGTCTGGC
pKT25-CcmA-F	CGG GGATCC GTGACAAATATAATTTCAGTAG
pKT25-CcmA-R	CCG GAATTC TTATACGAAGCGATAATCTAG
pKT25-CcmB-F	CGG GGATCC ATGAAAAGAGGCATCAGCT
pKT25-CcmB-R	CCG GAATTC TTAGTTAGTACTCACTCGCA
pKT25-CcmC-F	CGG GGATCC ATGTGGAAATGGTTACACCCTTA
pKT25-CcmC-R	CCG GAATTC TTATTTGACCTCCTCAGC

pKT25-CcmD-F	CGG GGATCC ATGCAATTCGATTCTATCA
pKT25-CcmD-R	CCG GAATTC TCACGATTTGTTACTCCTAG
pKT25-CcmE-F	CGG GGATCC GTGAACCCAAGACGCAAA
pKT25-CcmE-R	CCG GAATTC TTATTGAGTTGCCGATTT
pKT25-CcmF-F	CGG GGATCC ATGATCCCAGAACTTGGACA
pKT25-CcmF-R	CCG GAATTC TTATTGAGCGGTAGCTAATTTC
pKT25-CcmG-F	CGG GGATCC ATGATTCCAGAAATCGG
pKT25-CcmG-R	CCG GAATTC TCATTGGGTTACCGTCT
pKT25-CcmH-F	CGG GGATCC ATGAGAACACTGACAAA
pKT25-CcmH-R	CCG GAATTC TCATTTACTGTCACGCTT
pKT25-CcmI-F	CGG GGATCC ATGACGACATTTTGGATTTTTATTGC
pKT25-CcmI-R	CCG GAATTC TTATTGTACTTGAGTATCCAG
Complementation of homologues	CCG GAATTC
pHGEN-P*tac*-*Tc*TANGO2$^{\text{strep-tag-II}}$-F	C GAGCTC ATGTGCATATTATTTATCGCGATTG
pHGEN-P*tac*-*Tc*TANGO2$^{\text{strep-tag-II}}$-R	CGCGGATCCTTATTTTTCGAACTGAGGGTGAGACCATCCTTTCTGCGTCAATTCAA
pHGEN-P*tac*-*Pa*TANGO2$^{\text{strep-tag-II}}$-F	CCG CTCGAG ATGTGTCTGATTGCCTTCGA
pHGEN-P*tac*-*Pa*TANGO2$^{\text{strep-tag-II}}$-R	CGCGGATCCTTATTTTTCGAACTGAGGGTGAGACCAGCGCGGCTGAGTCG
pHGEN-P*tac*-*Dm*TANGO2$^{\text{strep-tag-II}}$-F	CCG GAATTC ATGTGCGTGATATTCTTTTGTGC
pHGEN-P*tac*-*Dm*TANGO2$^{\text{strep-tag-II}}$-R	CCCAAGCTTCTATTTTTCGAACTGAGGGTGAGACCAAACGCCGTTTTGAAAATC
pHGEN-P*tac*-*Sc*TANGO2$^{\text{strep-tag-II}}$-F	CCG CTCGAG ATGTGCATTTTAATGGCCACAA
pHGEN-P*tac*-*Sc*TANGO2$^{\text{strep-tag-II}}$-R	CGCGGATCCTTATTTTTCGAACTGAGGGTGAGACCACAATTTGAATTTAAACCTTTTTTCC
pHGEN-P*tac*-*At*TANGO2$^{\text{strep-tag-II}}$-F	CCG CTCGAG ATGTGCATAGCAGTATTTCTGTG
pHGEN-P*tac*-*At*TANGO2$^{\text{strep-tag-II}}$-R	CCCAAGTCCTTATTTTTCGAACTGAGGGTGAGACCAAATGCTTTGGTTTTGTATTATTACAAAC
pHGEN-P*tac*-*Hs*TANGO2$^{\text{strep-tag-II}}$-F	CCG CTCGAG ATGTGCATCATCTTCTTTAAGTTTGA
pHGEN-P*tac*-*Hs*TANGO2$^{\text{strep-tag-II}}$-R	CGCGGATCCTTATTTTTCGAACTGAGGGTGAGACCAGCTCTGCAGTGTGAACT
Biotinylation	
pHGEN-P*tac*-HtpA-BirA*-HtpA-F	CCG GAATTC ATGTGCATACTCTTCATCGCG
pHGEN-P*tac*-HtpA-BirA*-HtpA-R	TCTGGAGGTTCTGGAAAAAACCACTAAACAGTCTGGC
pHGEN-P*tac*-HtpA-BirA*-BirA*-F	ATGAAGGATAACACCGTGCC
pHGEN-P*tac*-HtpA -BirA*-BirA*-R	CGCGGATCCTTATTTTTCTGCACTACGCAGG
AP-PCR	
Run1seq	AGACCGGGGACTTA
Run1Arb	AAGAGTGAGACTGTAGCTAANNNNNNNNNNNNNNAAAAA
Run2seq	TTATCAGCCAACCTGT
Run2Arb	AAGAGTGAGACTGTAGCTAA
T-vector-F	GAGCGGATAACAATTTCACACAGG
T-vector-R	CGCCAGGGTTTTCCCAGTCACGAC
Promoter Activity Assay	
pHGI01-PHtpA-F	CCG GAATTC GCCTATAGCGGCTGGAG
pHGI01-PHtpA-R	CCC AAGCTT GTAATCATGGTCAT AGCAGGCTAAGTTTTTCCTAGG
pHGI01-PSO0127-F	CCG GAATTC AAATCGACGCGAATATCGGT
pHGI01-PSO0127-R	CCC AAGCTT GTAATCATGGTCAT TAACCCGCCTAAAGAGACG
pHGI01-P*hemA*-F	CCG GAATTC TGTGAGGGTGAGTTCGTCC
pHGI01-P*hemA*-R	CCC AAGCTT GTAATCATGGTCAT CTGACTCTTTTCTAGATTCTTGGC
pHGI01-P*hemC*-F	CCG GAATTC GCCGTGCTGCCATGGCAT
pHGI01-P*hemC*-R	CCC AAGCTT GTAATCATGGTCAT GCCTAGTTTCCATCCGAGATC
pHGI01-P*hemG*-F	CCG GAATTC GCAGTAACGGCTGTGTA
pHGI01-P*hemG*-R	CCC AAGCTT GTAATCATGGTCAT GGGGTATTCTCGTTCAGTAA
pHGI01-P*hemH*-F	CCG GAATTC CGATTGTGCTAAAGG
pHGI01-P*hemH*-R	CCC AAGCTT GTAATCATGGTCAT AAAAAGCCTGCTTAAGCAGG
