# Supplementary material for: Bacterial TANGO2 homologs are heme-trafficking proteins that facilitate biosynthesis of cytochromes c
Source: mBio. 2023 Jul 18;14(4):e01320-23. doi: 10.1128/mbio.01320-23 (PMC10470608; doi:10.1128/mbio.01320-23)
Supplement: Fig. S4 — SO0126 has no significant effect on heme levels. [file mbio.01320-23-s0004.pdf]

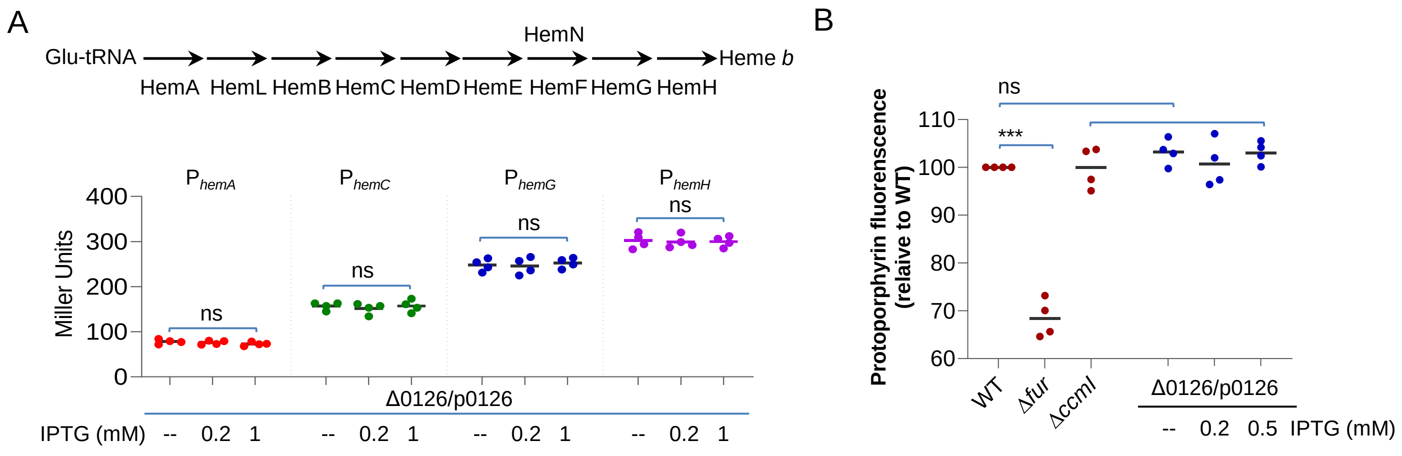

FIG S4. SO0126 has no significant effect on heme levels. (A) Effect of SO0126 on heme biosynthesis. Promoters for genes encoding four key enzymes in the heme biosynthesis pathway were assayed with integrative *lacZ* reporters in strains producing SO0126 at varying levels. β-Galactosidase activities were determined and are presented in Miller Units. (B) Relative heme *b* content assay. Protoporphyrin is the intermediate in the final step of the heme biosynthesis reflecting the amounts of heme. The content of protoporphyrin in WT is set to be 100%, and Δ*fur* which has low intracellular heme level is used as a negative control (69, 70).
